# Complex polar machinery required for proper chromosome segregation in vegetative and sporulating cells of *Bacillus subtilis*

**DOI:** 10.1111/mmi.13393

**Published:** 2016-05-18

**Authors:** Tomas G. Kloosterman, Rok Lenarcic, Clare R. Willis, David M. Roberts, Leendert W. Hamoen, Jeff Errington, Ling J. Wu

**Affiliations:** ^1^ Centre for Bacterial Cell Biology, Institute for Cell and Molecular Biosciences, Medical School, Newcastle UniversityNewcastle upon TyneUK; ^2^ Department of Molecular GeneticsGroningen Biomolecular Sciences and Biotechnology Institute, University of GroningenGroningenThe Netherlands; ^3^ Lek Pharmaceuticals d.d.MengesSlovenia; ^4^ Department of Cell Biology & PhysiologySwammerdam Institute for Life Sciences, University of AmsterdamAmsterdamThe Netherlands

## Abstract

Chromosome segregation is an essential process of cell multiplication. In prokaryotes, segregation starts with the newly replicated sister origins of replication, *oriCs*, which move apart to defined positions in the cell. We have developed a genetic screen to identify mutants defective in placement of *oriC* during spore development in the Gram‐positive bacterium *Bacillus subtilis*. In addition to the previously identified proteins Soj and DivIVA, our screen identified several new factors involved in polar recruitment of *oriC*: a reported regulator of competence ComN, and the regulators of division site selection MinD and MinJ. Previous work implicated Soj as an important regulator of *oriC* positioning in the cell. Our results suggest a model in which the DivIVA‐interacting proteins ComN and MinJ recruit MinD to the cell pole, and that these proteins work upstream of Soj to enable *oriC* placement. We show that these proteins form a polar complex, which acts in parallel with but distinct from the sporulation‐specific RacA pathway of *oriC* placement, and also functions during vegetative growth. Our study further shows that MinD has two distinct cell cycle roles, in cell division and chromosome segregation, and highlights that cell probably use multiple parallel mechanisms to ensure accurate chromosome segregation.

## Introduction

Chromosome segregation is one of the most fundamental processes in biology. However, details of the mechanisms responsible for accurately configuring and segregating bacterial chromosomes remain poorly resolved. Spore formation in the Gram‐positive bacterium *Bacillus subtilis* offers a particularly tractable system for studying chromosome organization and segregation (Errington, 2010; Possoz *et al*., 2012). Typical of the bacteria, *B. subtilis* has a single circular chromosome and a single fixed origin of replication (*oriC*). Bidirectional replication forks emanate from *oriC* and meet at the terminus, *terC*. The cells are haploid and replication is regulated so as to occur once per cell cycle, in parallel with cell growth (Toro and Shapiro, 2010).

**Figure 1:**
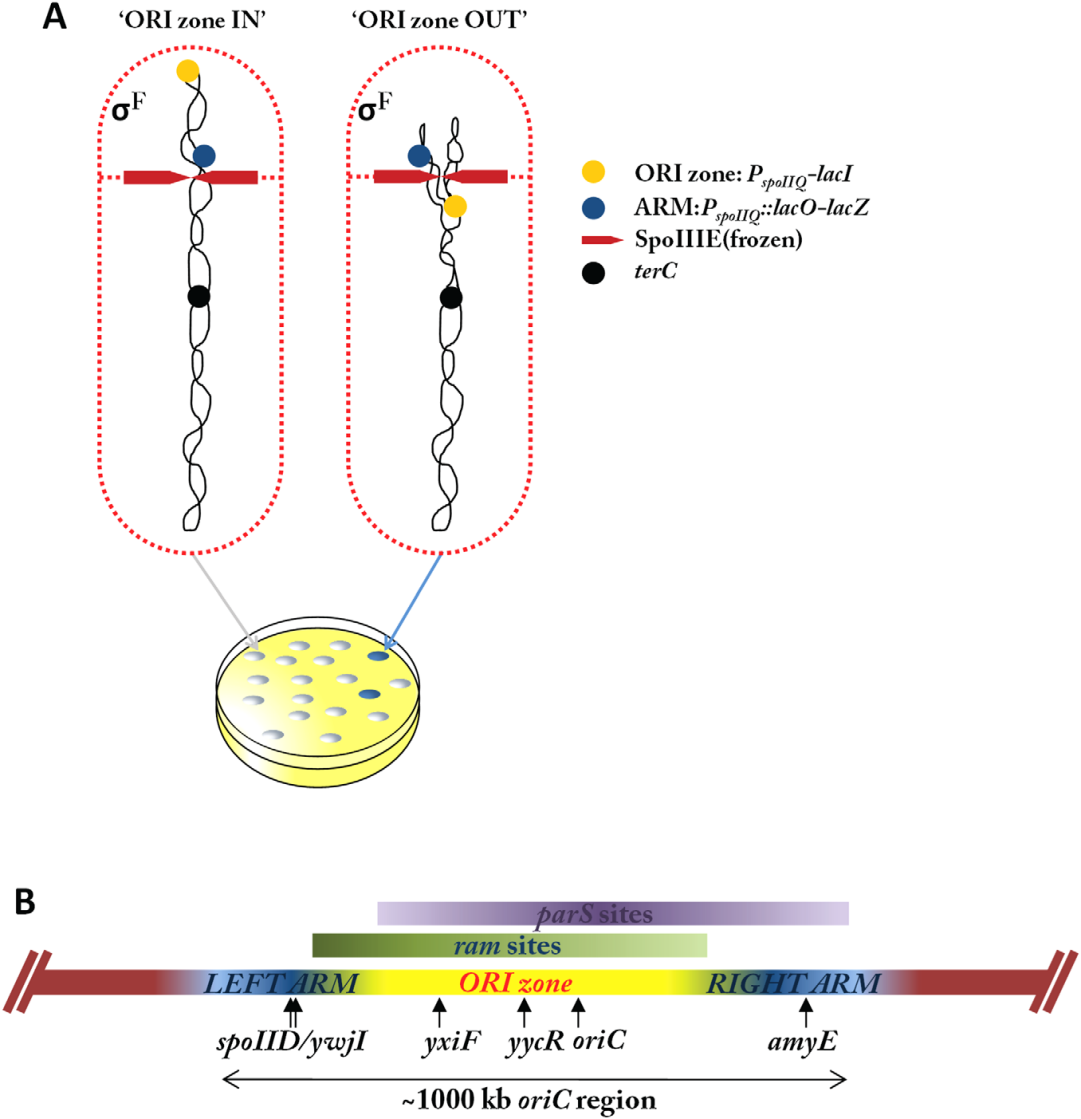
Organization of the *oriC* region and design of the genetic screen for mutants with an ‘ORI zone out’ phenotype. A. Schematic illustration of the compartmentalization that occurs during the early stages of sporulation, where asymmetric division leads to the generation of a small prespore containing only one third of a chromosome, and a larger mother cell. σ^F^ drives expression of the *spoIIQ* (*P_spoIIQ_*) gene exclusively in the prespore compartment. Expression of the *lacZ* reporter is induced by σ^F^ but this is overruled by repression by LacI via its operator *lacO*, when expressed in the prespore compartment from the *P_spoIIQ_*. SpoIIIE(frozen) (=SpoIIIE36) indicates the mutant form of SpoIIIE that does not allow DNA translocation into the prespore compartment. B. Diagram of the *oriC* region of the chromosome (horizontal bar) that is trapped in the prespore during the initial stage of spore formation, divided into the left and right ARM zones and the ORI zone according to the results of our prespore chromosome trapping assays. Regions enriched in RacA binding sites (*ram*, green) and Spo0J binding sites (*parS*, purple) are indicated above. Colour intensity roughly corresponds to the distribution with which the respective motifs occur in the DNA. Vertical arrows indicate the positions of the various genetic constructs used in the screen and for the prespore‐specific fluorescent reporter assay.

Endospore formation (hereafter, ‘sporulation’) is a complex adaptive response to starvation that is characteristic of the *Firmicutes* group of bacteria, largely those of the genera *Bacillus* and *Clostridium* (Al‐Hinai *et al*., 2015). During sporulation, the cell cycle is modified such that a cell with two and only two completely replicated chromosomes is generated prior to septation (Burkholder *et al*., 2001; Veening *et al*., 2009). Morphologically, it begins with a conformational change of the nucleoid to form an elongated structure called the axial filament (Hilbert and Piggot, 2004), followed by an asymmetric cell division, which divides the sporulating cell into two unequally sized cell compartments: the small prespore or forespore, which develops into a mature, dormant endospore and the larger mother cell, which lyses after contributing its resources to the development of the spore (Errington, 2010; Tan and Ramamurthi, 2014). The axial filament extends from pole to pole, with the two *oriC* regions of the sister chromosomes associated with opposite cell poles (Ben‐Yehuda *et al*., 2003; Wu and Errington, 2003) and the two *terC* regions located close to each other at about mid cell (Fig. 1A). Chromosomal loci between these sites are arranged in a linear order between poles and mid cell, reflecting their relative positions on the chromosome ‘left’ and ‘right’ arms (Webb *et al.*, 1997; Teleman *et al*., 1998; Wu and Errington, 1998; Wang *et al*., 2014). The sporulation division septum, which is located very close to one of the cell poles, closes around the axial filament in such a way that initially only about 30% of the chromosome destined for the spore is located inside the small prespore compartment, while the mother cell houses the remaining 70% of this chromosome, together with the second chromosome that belongs to the mother cell (Fig. 1A) (Wu and Errington, 1994; Wu *et al*., 1995; Wu and Errington, 1997). A widely conserved protein, SpoIIIE (FtsK) (Sherratt *et al*., 2010), then translocates the remaining segment of the chromosome into the prespore compartment to complete the segregation process (Wu and Errington, 1994; Bath *et al*., 2000). The prespore and the mother cell subsequently undergo a complex differentiation process that is initiated by activation of a specialized RNA polymerase sigma subunit, σ^F^, in the prespore compartment (Fig. 1A) (Haldenwang, 1995; Londoño‐Vallejo *et al*., 1997; Iber *et al*., 2006). While the prespore and the mother cell ultimately each receive one intact chromosome, the transient genetic asymmetry that exists prior to the completion of chromosome translocation is crucial for the activation of σ^F^ in the prespore and the establishment of compartment‐specific programmes of transcription (Fransden *et al*., 1999).

The DNA translocase SpoIIIE is located at the leading edge of the asymmetric septum (Wu and Errington, 1997; Fleming *et al*., 2010; Fiche *et al*., 2013) (Fig. 1A). Certain mutations in the *spoIIIE* gene abolish DNA translocation activity but enable assembly of a stable complex with the DNA enclosed by the constricting septum (Wu and Errington, 1994). Although in these cells the chromosomes are ‘frozen’ in an asymmetric state, with 70% of the prespore chromosome stuck in the mother cell, σ^F^ is correctly activated in the small prespore compartment and it can turn on σ^F^‐dependent genes if those genes are on the segment of DNA that locates inside the prespore (Wu and Errington, 1994; Wu *et al*., 1995; Wu and Errington, 1997). A ‘*spoIIIE* trapping assay’ based on σ^F^‐dependent reporters in a transfer‐inactive *spoIIIE* mutant has been used extensively to probe the segment of DNA initially trapped in the prespore compartment, as well as the factors required for chromosome orientation and configuration in the early stages of sporulation (Wu and Errington, 1998; Wu and Errington, 2002). It is now known that the DNA segment that is already in the prespore compartment when the asymmetric septum forms centres slightly to the left of *oriC* (Fig. 1B) (Wu and Errington, 1998; Wu and Errington, 2002), and that many factors are involved in ensuring correct chromosome configuration and establishing the interaction between the DNA segment and the cell pole, including sporulation‐specific RacA (Ben‐Yehuda *et al*., 2003; Wu and Errington, 2003), and vegetatively expressed DivIVA (Thomaides *et al*., 2001; Perry and Edwards, 2004), Spo0J (ParB) (Sharpe and Errington, 1996; Wu and Errington, 2002) and Soj (ParA) (Wu and Errington, 2002; Sullivan *et al*., 2009).

DivIVA is a conserved protein that is involved in a wide range of processes associated with cell pole development in Gram‐positive bacteria (Vicente and Garcia‐Ovalle, 2007; Hempel *et al*., 2008; Strahl and Hamoen, 2012). This peripheral membrane protein is targeted to regions with specific curvature, particularly the base of a septum (Edwards and Errington, 1997; Lenarcic *et al*., 2009). In *B. subtilis*, DivIVA is the topological determinant of the Min system involved in division site placement during vegetative growth (Edwards and Errington, 1997; Marston *et al*., 1998), and in sporulation it acts as an important factor in polar chromosome attachment (Thomaides *et al*., 2001; Perry and Edwards, 2004; Perry and Edwards, 2006). The two functions are separable (Thomaides *et al*., 2001) and, in a *divIVA* mutant that specifically affects chromosome segregation, the prespore chromosome is trapped in an unusual configuration in which the *oriC* region (‘ORI zone’; perhaps 200 kbp or so of the *oriC*‐proximal DNA, Fig. 1A and B) lies outside the prespore compartment, while flanking regions either side of the ORI zone (left and right ARM zones; Fig. 1B) are correctly placed (Wu and Errington, 2003). Exclusion of the ORI zone from the prespore has also been reported for the *soj* null mutant, though the defect is much milder (Sullivan *et al*., 2009). Finally, RacA was identified as a sporulation‐specific factor required for accurate prespore chromosome segregation (Ben‐Yehuda *et al*., 2003; Wu and Errington, 2003; Ben‐Yehuda *et al*., 2005). It is recruited to the cell poles by DivIVA and it binds to multiple RacA‐binding (*ram*) sites that are located mainly in a 250 kbp region to the left of *oriC* (Fig. 1B). Surprisingly, although loss of RacA also resulted in a defect in chromosome segregation, the phenotype was different from that of the *divIVA* mutant: about half of the prespores failed to capture any DNA, and the other half of the cells had the correct *oriC* segment of the chromosome (Ben‐Yehuda *et al*., 2003; Wu and Errington, 2003).

The *soj* (*parA*) and *spo0J* (*parB*) genes encode well known, widely conserved factors originally identified through their roles in the stable maintenance of low copy number plasmids (Møller‐Jensen *et al*., 2000; Surtees and Funnell, 2003; Mierzejewska and Jagura‐Burdzy, 2012). They are now known to be present in most bacterial genomes (with the curious exception of *E. coli* and close relatives), and thought likewise to be involved in chromosome segregation. Mutations in *soj* and/or *spo0J* affect proper capturing of the prespore chromosome in the *spoIIIE* trapping assay (Sharpe and Errington, 1996; Wu and Errington, 2003; Sullivan *et al*., 2009). During vegetative growth, these proteins not only are required for proper chromosome segregation (Lee *et al*., 2003; Lee and Grossman, 2006; Gruber and Errington, 2009), but also contribute to regulation of the initiation of DNA replication and, indirectly, to the initiation of sporulation (Murray and Errington, 2008; Veening *et al*., 2009). Spo0J is a site‐specific DNA binding protein that binds to several *parS* sites, located mainly around the *oriC* region (Fig. 1B) (Breier and Grossman, 2007). It spreads from primary binding sites by a mechanism that probably involves direct lateral protein–protein interactions as well as bridging or looping (Murray *et al*., 2006; Graham *et al*., 2014). ParB‐*parS* complexes can recruit bacterial condensin (ScpAB‐SMC), which is important for chromosome organisation and segregation, at least in vegetative cells (Gruber and Errington, 2009; Sullivan *et al*., 2009; Gruber *et al*., 2014). Soj is a dimeric ATPase that in some organisms has been shown to participate in active movement of *parS*‐*oriC* complexes (Ptacin *et al*., 2010; Schofield *et al*., 2010; Lim *et al*., 2014). Its ATPase activity can be stimulated by a short N‐terminal peptide of Spo0J, and different forms (ADP or ATP‐bound, monomeric or dimeric) of *B. subtilis* Soj can interact with Spo0J‐DNA (Scholefield *et al*., 2011) and the DNA replication initiation protein DnaA (Murray and Errington, 2008). Perturbation of this latter interaction can lead to a defect in the initiation of sporulation, which resulted in the original naming of *spo0J* (Ireton *et al*., 1994; Veening *et al*., 2009).

To dissect further the mechanism of prespore chromosome segregation, we developed a screen for mutations that generate a *divIVA*‐like ‘ORI zone out’ phenotype (9). Characterization of a large collection of transposon mutants with the required phenotype identified two genes, *minD* and *comN*, that have other, unrelated functions in vegetative growth, and defined a multicomponent polarly‐located machinery required for correct placement of the *oriC* region in *B. subtilis* during spore development as well as in vegetative cells.

## Results

### A screen for mutations affecting *oriC* to pole recruitment during sporulation

The basis of the transposon screen is illustrated in Fig. 1A. We built two strains, one carrying a *lacZ* reporter gene located in the right ARM zone (*amyE*, +327 kbp clockwise from *oriC* on the 4218 kbp chromosome) and the other in the left (*spoIID*, −438 kbp; see also Fig. 1B). The *lacZ* reporter was driven by a prespore‐specific σ^F^‐dependent promoter, *P_spoIIQ_* (Londoño‐Vallejo *et al*., 1997), which was modified to be regulated negatively by LacI. The *lacI* gene was expressed from an unmodified *P_spoIIQ_* promoter and was located in the ORI zone (−192 kbp). In an otherwise wild‐type [but *spoIIIE*(frozen)] background, the *lacZ* reporter gene would not be expressed significantly, because it occupies the same compartment as the *lacI* gene, giving white colonies on X‐Gal plates unless IPTG was included in the medium (Supporting Information Fig. S1). Mutants giving an ORI zone out phenotype would give blue colonies because the *lacI* repressor gene is excluded from the prespore. In all the subsequent experiments involving σ^F^‐dependent reporters, the strains always carried a *spoIIIE*(frozen) mutation (*spoIIIE36*), unless stated otherwise.

We used random transposon mutagenesis to look for mutants giving blue colonies upon sporulation on Nutrient Agar plates. In a series of independent experiments we screened over 100,000 colonies and characterized over 100 mutants. DNA sequencing from one of the transposon ends revealed the sites of insertion. We found one hit upstream of *divIVA* (transposon insertion at 51 bp upstream of the start codon of the *divIVA* ORF), which was expected. We assume that this affects the level of expression of the gene: we did not expect to obtain mutations elsewhere in *divIVA* because null mutants have poor viability (Edwards and Errington, 1997; Thomaides *et al*., 2001). Two hits were found close to the gene encoding the histone‐like protein Hbsu (Klein and Marahiel, 2002), which probably have a global effect on chromosome organization and were not further investigated at this stage. Several hits were in the *soj* gene, which again was expected. Surprisingly, multiple hits were also found in the *minD* gene. MinD is the activator of the cell division inhibitor MinC (de Boer *et al*., 1990; Marston and Errington, 1999b). The MinCD complex is recruited to the division site by DivIVA, through MinJ, to prevent divisions near the nascent septum and the cell poles during vegetative growth (Bramkamp *et al*., 2008; Patrick and Kearns, 2008; Bramkamp and van Baarle, 2009; van Baarle *et al*., 2013). Absence of MinC or MinD often results in cells dividing near the cell pole to produce a tiny, DNA‐less cell called a minicell (Reeve *et al*., 1973; de Boer *et al*., 1990; Marston and Errington, 1999b). The MinCD complex has previously been implicated in the regulation of SpoIIIE assembly, at the leading edge of the asymmetric septum during sporulation (Sharp and Pogliano, 2002), but we believed this did not explain the present results for reasons that will become clear below. Finally, multiple hits were also found in a gene called *comN (yrzD)*. ComN is reported to be involved in post‐translational regulation of the *comE* competence operon (Ogura and Tanaka, 2009), but had not previously been implicated in chromosome segregation. Interestingly, localization of ComN is also influenced by DivIVA (dos Santos *et al*., 2012). Of note, multiple hits in *soj*, *comN* and *minD* were obtained independent of whether the *lacZ* gene was located in the right or the left ARM region.

### *comN* and *minD* mutants are both defective in *oriC* trapping during sporulation

**Figure 2:**
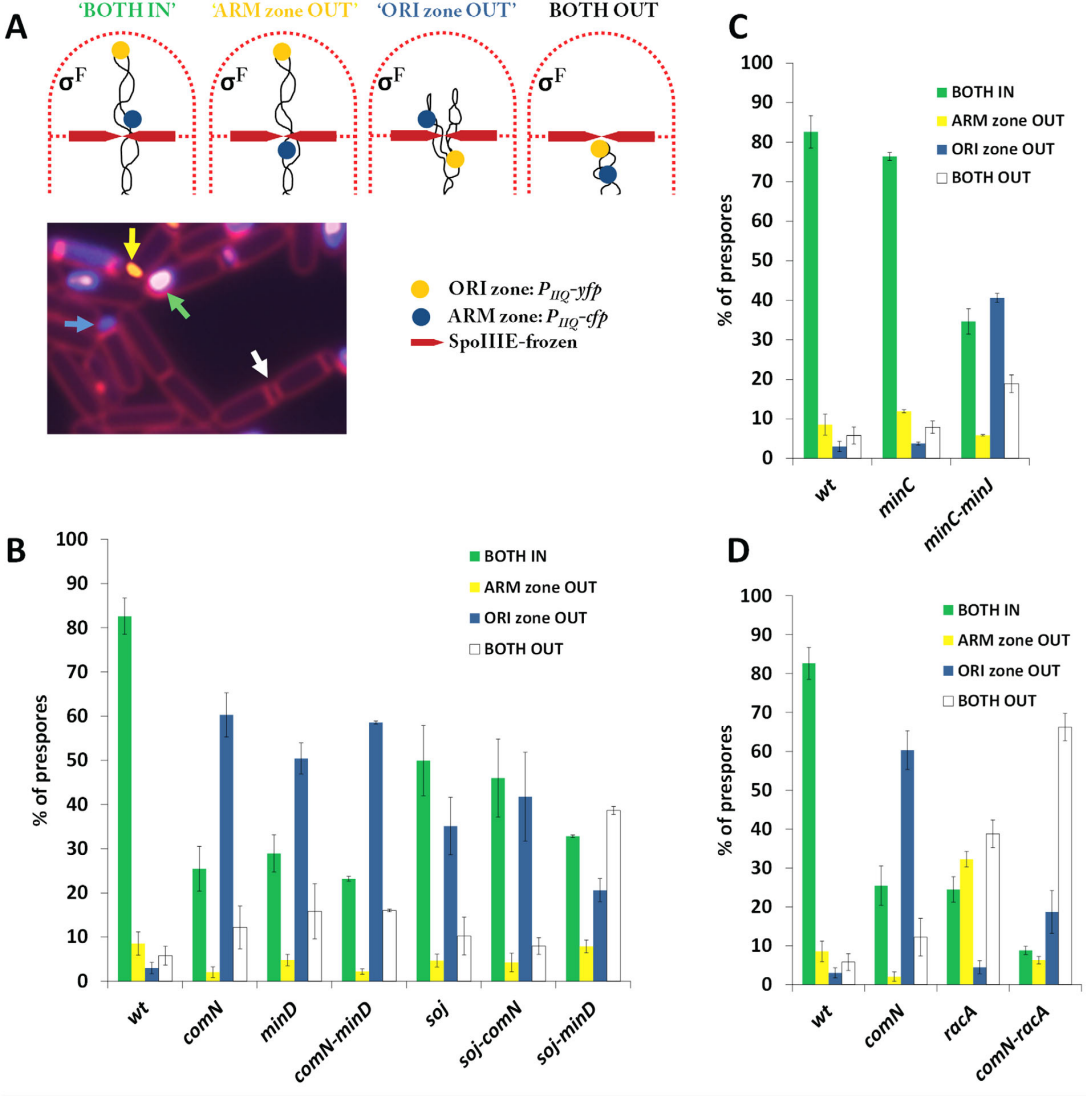
Use of a *P_spoIIQ_*‐based fluorescent reporter assay to quantify trapping of ORI and ARM zones in the prespore compartment of the mutant strains indicated in the graphs. A. Principle of the assay as developed previously by (Sullivan *et al*., 2009). The prespore compartment contains either both the ORI and ARM zones (green ‘BOTH IN’), only the ORI zone (yellow ‘ARM zone OUT’), only the ARM zone (blue ‘ORI zone OUT’), or neither of these (black ‘BOTH OUT’). The ORI zone is represented by a *P_spoIIQ_‐yfp* construct inserted at *yycR* (−79 kbp; yellow spot) and the ARM zone is represented by a *P_spoIIQ_‐cfp* construct inserted at *ywjI* (−418 kbp; blue spot). The microscopic image below (*comN* mutant and *spoIIIE(frozen)* mutant background) illustrates prespores of all 4 classes indicated by arrows with corresponding colours, that is, green=both reporters in the prespore, yellow=only the ORI zone (YFP) reporter in the prespore, blue=only the ARM zone reporter, white=no reporter in the prespore (CFP). Cell membranes were stained with the FM5‐95 dye. B. ORI zone out phenotypes of *comN*, *minD* and *soj* mutants quantified by the fluorescent reporter assay as described in A. C, D. Chromosome trapping phenotypes of the *minC*, *minJ* (C) and *racA* (D) mutants as assessed by the fluorescent reporter assay described in A. Cells were allowed to sporulate for 3 h before microscopic imaging. At least 300 cells were counted for each strain in each experiment. The values are averages of at least three independent experiments. Error bars represent standard deviations. For the wild‐type, data were averaged among all experiments and therefore, the same numbers are shown in panels B–D.

To verify our screening results, we constructed an in‐frame marker‐free deletion mutant of *comN* and confirmed its phenotype of *oriC* exclusion from the prespore. The defect could be complemented with *P_comN_‐comN* expressed ectopically at *amyE* (Supporting Information Fig. S2). This showed that the defect of the *comN* mutant in *oriC* recruitment to the cell pole was not caused by a polar effect. For reasons we do not understand, although a *comN* mutant had previously been shown to have a 200‐fold reduction in competence for genetic transformation (Ogura and Tanaka, 2009), in our experiments *comN* mutants could be as readily transformed with chromosomal DNA as the wild‐type strain. We next examined the organisation of the chromosome quantitatively in a *comN* null mutant and a *minD* mutant using a prespore‐specific fluorescent reporter system based on the ‘*spoIIIE* trapping assay’ (Sullivan *et al*., 2009) (Fig. 2A). This system makes use of a *cfp* reporter gene located in the ARM zone (*ywjI*, −418 kbp, see also Fig. 1B) and a *yfp* reporter gene within the ORI zone (*yycR*, −79 kbp), both controlled by *P_spoIIQ_*, which is active only in the prespore. As expected, 3 h after the induction of sporulation, prespores of the wild‐type contained mostly signals from both reporters (>80%; green bar in Fig. 2B), showing that both the ORI and the ARM zones were inside the prespore. In line with the genetic screen, the *comN* deletion mutant displayed a strong specific defect in trapping of the ORI zone marker in the prespore, with only about 1/4 of the sporulating cells showing signals from both reporters: the majority of cells (∼60%) had only the signal from the reporter in the ARM zone (blue bar in Fig. 2B). A similar pattern was seen when the ARM zone marker was on the right (*amyE*; +327 kbp) (Supporting Information Fig. S3).

Interestingly, although the *minD* mutant was more difficult to work with because of its high frequency of minicell production, it seemed to behave indistinguishably from the *comN* mutant, with ∼80% of the prespores showing the signal for the ARM zone (green and blue bars in Fig. 2B) but most of these (over 50%) did not have the ORI zone signal (blue bar in Fig. 2B).

To probe the positioning of the ORI zone directly, we used the *tetO*‐TetR fluorescent repressor operator system (FROS) with a *tetO* array inserted close to *oriC* (*yycR*, −79 kbp) that can be visualized by binding of TetR‐GFP expressed from *amyE* (+327 kbp) (Wagner *et al*., 2009) (Supporting Information Fig. S4A). In the *comN* mutant, approximately 60% of the cells had both *oriC* foci in the mother cell compartment, whereas almost all (>98%) wild‐type cells had one *oriC* in the prespore and one in the mother cell (Supporting Information Fig. S4B). Notably, in a *spoIIIE^+^* background, which did not lead to the ‘frozen’ state of the prespore chromosome, the *comN* mutant still mislocalized 35% of the *oriCs versus* <2% for the wild‐type (Supporting Information Fig. S4C). Again, the *minD* mutant had a very similar phenotype to that of the *comN* mutant (Supporting Information Fig. S4B and C). This result lent support to the *P_spoIIQ_‐cfp/yfp*‐based reporter assays and confirmed that in the absence of ComN or MinD the ORI zone is excluded from the prespore in a large proportion of the sporulating cells.

### *minJ* but not *minC* is also required for correct positioning of *oriC* in the prespore

Both MinD and DivIVA are members of the Min system that spatially regulates division septum placement, in which the cell division inhibitor MinC forms a complex with MinD to inhibit FtsZ assembly near cell poles and nascent septa (Marston *et al*., 1998; Marston and Errington, 1999b; Gregory *et al*., 2008). The complex is recruited to DivIVA through a fourth protein, MinJ, an integral membrane protein that bridges DivIVA and MinD (Bramkamp *et al*., 2008; Patrick and Kearns, 2008). To see whether MinC and MinJ were also required for *oriC* positioning at the cell pole during sporulation, we examined trapping of the ORI and ARM markers as described above in mutants of *minC* and *minJ*. We tested a single, non‐polar *minC* mutant and were surprised to obtain essentially wild‐type level of chromosome trapping (Fig. 2C and Supporting Information Fig. S4C), indicating that perturbation of cell division through loss of Min function is not the reason for the observed trapping defect of the *minD* mutants, and therefore that this is a separate additional function of MinD.

Mutants of *minJ* have a strong MinCD‐dependent cell division defect (Bramkamp *et al*., 2008; Patrick and Kearns, 2008). To be able to test the involvement of MinJ in *oriC* placement we introduced a *minC* mutation into the *minJ* mutant, and compared this with a *minC* single mutant. As shown in Fig. 2C, the *minJ* mutation, similarly to the *comN* and *minD* mutations, also severely affected trapping of the ORI but not the ARM zone in the prespore. Therefore, MinJ is also required for *oriC* positioning, potentially through its role in MinD recruitment. We assume that *minJ* mutations were not picked up in the screen because of the growth and division defect in *minJ* single mutants, just as for *divIVA*.

### ComN, MinD and Soj act in the same pathway in *oriC* positioning

Since deletion of *comN*, *minD* and *soj* all cause the *oriC* proximal marker specifically to be excluded from the prespore (Fig. 2B) (Sullivan *et al*., 2009), we reasoned that these three proteins act in a common pathway that governs *oriC* positioning. To test this we first combined *comN* and *minD* mutations and found that the double mutant exhibited a similar ORI zone trapping defect as the single mutants, consistent with ComN and MinD acting in the same pathway (Fig. 2B). We then examined the effects of a *soj* mutation, which has a slightly milder trapping defect than the *comN* or *minD* deletion (Fig. 2B). Interestingly, although double mutants of *comN soj* and *minD soj* both exhibited an ORI zone trapping defect, it was relatively mild, of similar magnitude to that of the *soj* single mutant in terms of reduction in cells with both markers correctly trapped (Fig. 2B). Similar results were obtained with the direct trapping assay using the *tetO*/TetR system (Supporting Information Fig. S4C).

### ComN and MinD act in an *oriC*‐trapping pathway separate from that of RacA

We previously showed that *racA* and *soj* mutations act synergistically and that the double mutant has a much stronger defect in *oriC* trapping than either single mutant (Wu and Errington, 2003). The action of RacA is quite well established and its binding (*ram*) sites on the chromosome mainly lie to the left of *oriC* (Fig. 1B) (Ben‐Yehuda *et al*., 2005). Using the *P_spoIIQ_*‐based fluorescent reporter system we found that, in contrast to ComN and MinD, RacA predominantly affects trapping of the ARM zone (Fig. 2D). When the *racA* deletion was combined with the *comN* mutation, a more severe defect was also obtained and most prespores were devoid of both the CFP and YFP signals during sporulation (Fig. 2D), similar to our previously reported results with *racA* and *soj* (Wu and Errington, 2003). Consistent with the trapping assay, the sporulation efficiency of the *comN racA* double mutant was also greatly reduced when compared with the single mutants (Supporting Information Table S3). All these results support the idea that there are at least two distinct mechanisms governing the positioning of the *oriC* region of the chromosome during sporulation; one dependent on RacA and the other on ComN, MinD and Soj.

### The *comN*/*minD* defect affects *oriC* positioning before asymmetric septation

**Figure 3:**
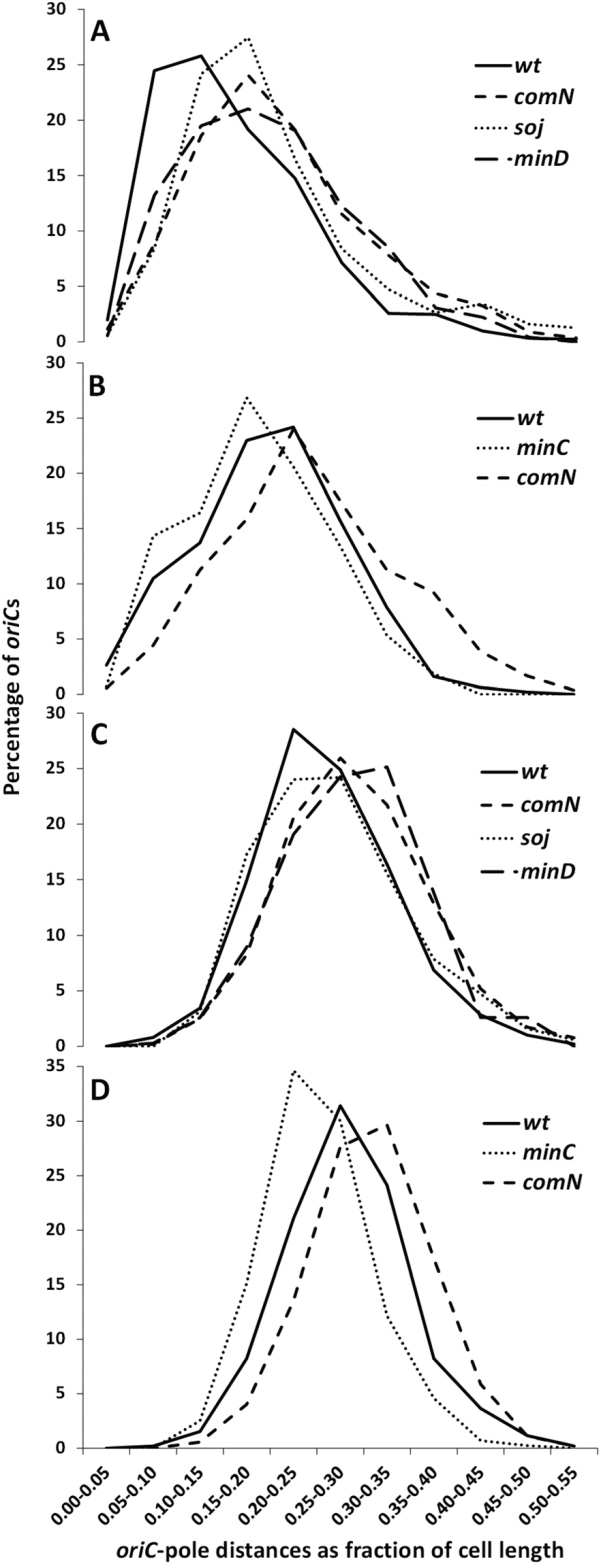
Distribution of *oriC*‐pole distances in the indicated strains in sporulation conditions (A, B) and during mid‐logaritmic vegetative growth in CH medium (C, D). As a direct marker of *oriC* localization TetR‐GFP (expressed from *amyE*, +327 kbp) bound to an *oriC* proximal *tetO_25_* array (inserted at *yycR*, −79 kbp) was used. Cells were stained with membrane dye FM5‐95 to reveal the cell poles. The numbers of cells measured for the *oriC*‐pole distances per strain/condition ranged from 432 to 1252. For sporulation conditions (A, B), cells were grown for approximately 90 min in sporulation medium. At this stage many cells have formed an axial filament but only very few cells contained a sporulation septum. Cells contained the wild‐type copy of the *spoIIIE* gene.

To test when mislocalization of *oriC* occurs, we measured the distance between the *oriC* (TetR‐GFP) foci and the cell pole (as a fraction of cell length) in *minD*, *comN* and *soj* mutants just before asymmetric septation commences. As shown in Fig. 3A, this distance was indeed significantly greater in the mutants than in wild‐type cells. *minD* mutants, and to a lesser extent *comN* mutants, have an altered length distribution compared to the wild‐type (Bramkamp *et al*., 2008) (Supporting Information Fig. S5C). To rule out an effect of cell length, *oriC*‐pole distances in cells of the same length class (Supporting Information Fig. S5A) were measured in the *comN* mutant and the wild‐type. This again showed a similar shift in the distribution of *oriC*‐pole distances (Supporting Information Fig. S5B). Of note, the efficiency of prespore formation in the *comN* mutant over the course of 3 h after induction of sporulation was very similar to the wild‐type strain, that is, at 100, 125 and 180 min after induction of sporulation the percentages of cells sporulating were 3%, 8% and 52% in the wild‐type and 3%, 12% and 55% in the *comN* mutant, respectively. As a further control, we then examined cells of a *minC* mutant, which has a very similar cell length distribution as *minD* mutants (Bramkamp *et al*., 2008). As shown in Fig. 3B, the distribution of *oriC*‐pole distance of the *minC* mutant was similar to that of the wild‐type. Thus, the *comN*, *minD* and *soj* mutants are all defective in sub‐polar positioning of the *oriC* region before the asymmetric septum forms.

### *comN* and *minD* mutants are affected in *oriC* positioning in vegetative cells

Since MinD and ComN are expressed throughout growth and sporulation, unlike RacA which is sporulation specific (Ben‐Yehuda *et al*., 2003; Wu and Errington, 2003), we wondered whether these factors might also have a role in *oriC* positioning during vegetative growth. Indeed, although neither of the mutants had an overt chromosome segregation defect, *oriC*‐pole distances were significantly greater in *minD* and *comN* mutant cells growing vegetatively compared to wild‐type cells (Fig. 3C). Curiously, no such effect was detectable for cells of a vegetatively growing *soj* mutant (but note that the *soj* mutant has a milder effect on *oriC* positioning in sporulating cells than *comN* or *minD* mutants; Fig. 3C). Again, the increased distances of *oriC* from the cell pole in vegetatively growing cells of *minD* and *comN* mutants were not a result of minicell production and/or increased cell length, because a *minC* mutant, which had a very similar minicell phenotype and cell length distribution to those of *minD* mutants, did not have an increased *oriC*‐pole distance (Fig. 3D). In fact, the distances were in general slightly reduced when compared to the wild‐type.

### ComN is required for proper localization of MinD but not MinC

It has previously been shown that localization of ComN to division septa requires DivIVA but not MinJ or MinD (dos Santos *et al*., 2012). To further study the role of ComN in chromosome segregation, we constructed a ComN‐GFP fusion expressed ectopically at *amyE* from its native promoter and checked that this was functional as judged by complementation of the *oriC* trapping defect of a *comN* null mutant (Supporting Information Fig. S2). In accordance with the previous study (dos Santos *et al*., 2012), we confirmed that ComN localizes to the septum and cell poles, and that this localization is dependent on DivIVA but not MinD, MinC or MinJ (Supporting Information Fig. S6).

**Figure 4:**
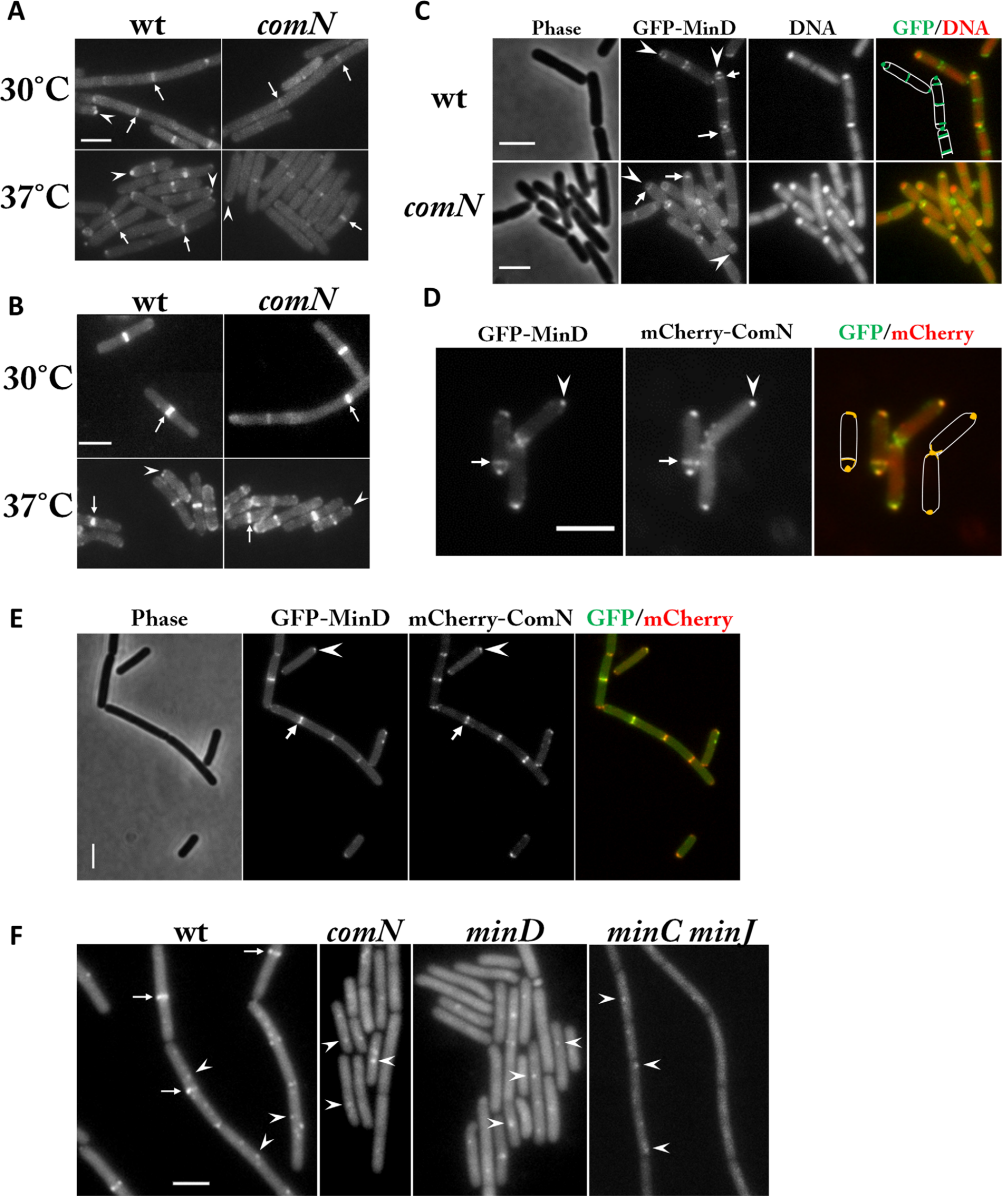
Septal/polar localization of GFP‐MinD and GFP‐Soj is dependent on ComN. A. Localization of GFP‐MinD expressed from the native locus (JAG118 from Gregory *et al*., 2008) in the wild‐type (left panels) and the *comN* mutant (right panels) during vegetative growth in CH medium at 30°C (upper panels) and 37°C (lower panels). B. Localization of GFP‐MinC expressed from the native locus (EBS499 from Gregory *et al*., 2008) in the wild‐type (left panels) and the *comN* mutant (right panels) during vegetative growth in CH medium at 30°C (upper panels) and 37°C (lower panels). C. Localization of GFP‐MinD expressed from the native locus (unpublished construct by Wu LJ, Schneeweiss J and Errington J) in the wild‐type (upper panels) and the *comN* mutant (lower panels) 85 min after the induction of sporulation. DNA was stained with DAPI. The right hand panels are merged images of the GFP and DNA images showing the positions of the MinD signals in relation to the chromosomes. The green colour in the cartoon in the upper right hand panel indicates the cellular positions of fluorescent signals from GFP‐MinD at (asymmetric) septa and cell poles. Outlines of the cells are in white. D, E. Colocalisation of GFP‐MinD and mCherry‐ComN during sporulation (D) as well as during vegetative growth (E). For, D, the yellow colour in the cartoon in the most right panel indicates the cellular positions of fluorescent signals from GFP‐MinD and mCherry‐ComN at an asymmetric septum and at cell poles. Outlines of the cells are in white. F. Localization of GFP‐Soj expressed from the native locus in the wild‐type and in *comN*, *minD* and *minC minJ* mutant strains during vegetative growth at 30°C in CH medium. For A–F, arrows point to (asymmetric) septa, and arrowheads indicate cell poles (Fig. A–E) or cytoplasmic foci (Fig. 4F). Scale bars represent 3 µm.

We then carried out the reciprocal experiments examining the effects of *comN* deletion on the localization of GFP fusions to MinD and MinC (Fig. 4A–E). Two different GFP‐MinD constructs were examined, and in both cases substantial effects on localization were observed. In particular, the prominent fluorescent bands associated with nascent division septa (arrows in Fig. 4A), and the foci at the poles (arrowheads in Fig. 4A) were greatly reduced in intensity or abolished in vegetative cells (Fig. 4A). Western blot analysis revealed no difference in the levels of MinD between the *comN* mutant and the wild type strain (data not shown). Thus, recruitment of MinD to the division site and retention at the cell pole is at least partially dependent on ComN. Surprisingly, localization of a MinC‐GFP fusion was not visibly affected by deletion of *comN* and most division sites had prominent fluorescent bands irrespective of whether or not the cells had a functional *comN* gene (Fig. 4B).

To test how much Min function was affected by the delocalization of MinD in the absence of ComN, we examined the number of minicells produced by the mutants and found that the *comN* mutant indeed produced a small number of minicells (1.5% minicells (*n* = 1842) vs. 0.3% (*n* = 1450) in the wild‐type), but the number was far less than that of the *minD* mutant (25% minicells, *n* = 291). It has been shown that in *B. subtilis* MinD is present in excess of MinC (sixfold to ninefold) (Muntel *et al*., 2014). It is therefore possible that the residual MinD localization in the absence of *comN* is sufficient to recruit MinC and restrict division to the mid cell site.

In the absence of the nucleoid occlusion protein, Noc, Min function becomes crucial for cell viability ‐ *noc min* double mutants are conditionally lethal at temperatures higher than 37°C (Wu and Errington, 2004). We reasoned that if ComN affects mainly the *oriC* segregation but not the division function of MinD, *comN noc* double mutants should be viable at 48°C. Indeed, a *comN noc* double mutant was able to grow at 48°C, whereas a *minD noc* double mutant was not (data not shown), consistent with the above conclusion that MinD has separable functions and that the cell division function of MinD in vegetative growth is largely unaffected in the absence of ComN.

### ComN and MinD colocalize at cell poles in sporulation

The involvement of ComN and MinD in the polar placement of the *oriC* region of the chromosome during spore development, and the dependence of MinD localization on ComN during growth predicted that the two proteins colocalized at cell poles during sporulation. Indeed, both MinD and ComN could be seen at the asymmetric septum and also as a focus at the cell pole (arrows and arrowheads, respectively, in Fig. 4C and D). Importantly, although weak bands of GFP‐MinD were still present at the asymmetric septa in the *comN* mutant, the polar foci of GFP‐MinD were effectively absent, and instead a faint, uniform signal could sometimes be seen all over the membrane surrounding the prespore, probably due to its general affinity for the membrane (Fig. 4C). To test the colocalization, we constructed a strain carrying fusions expressing both GFP‐MinD and mCherry‐ComN. As shown in Fig. 4D and E, GFP‐MinD colocalized with mCherry‐ComN at septa as well as at the poles in both sporulating and vegetative cells, consistent with the idea that ComN and MinD are present in the same functional complex.

### ComN, like MinD, is required for septal recruitment of Soj

Soj protein has a complex localization pattern, showing association with the nucleoid, the division septum, DnaA foci and Spo0J foci (Marston and Errington, 1999a; Quisel *et al*., 1999; Murray and Errington, 2008). We previously showed that septal recruitment of Soj depends on MinD (Murray and Errington, 2008), and based on the results described above it might also depend on ComN. In wild‐type cells, GFP‐Soj was localized to division septa (arrows in Fig. 4F) and as foci in the cytoplasm (arrowheads in Fig. 4F), as previously reported (Murray and Errington, 2008). In the *comN* deletion mutant, however, the septal bands of Soj were absent (Fig. 4F). Thus, ComN is, like MinD (Fig. 4F) (Murray and Errington, 2008), required for septal localization of Soj. Consistent with the hierarchy of interactions described above, GFP‐Soj localization at the cell poles was also dependent on MinJ (Fig. 4F).

### A network of interactions between the proteins required for ORI zone segregation during sporulation

**Figure 5:**
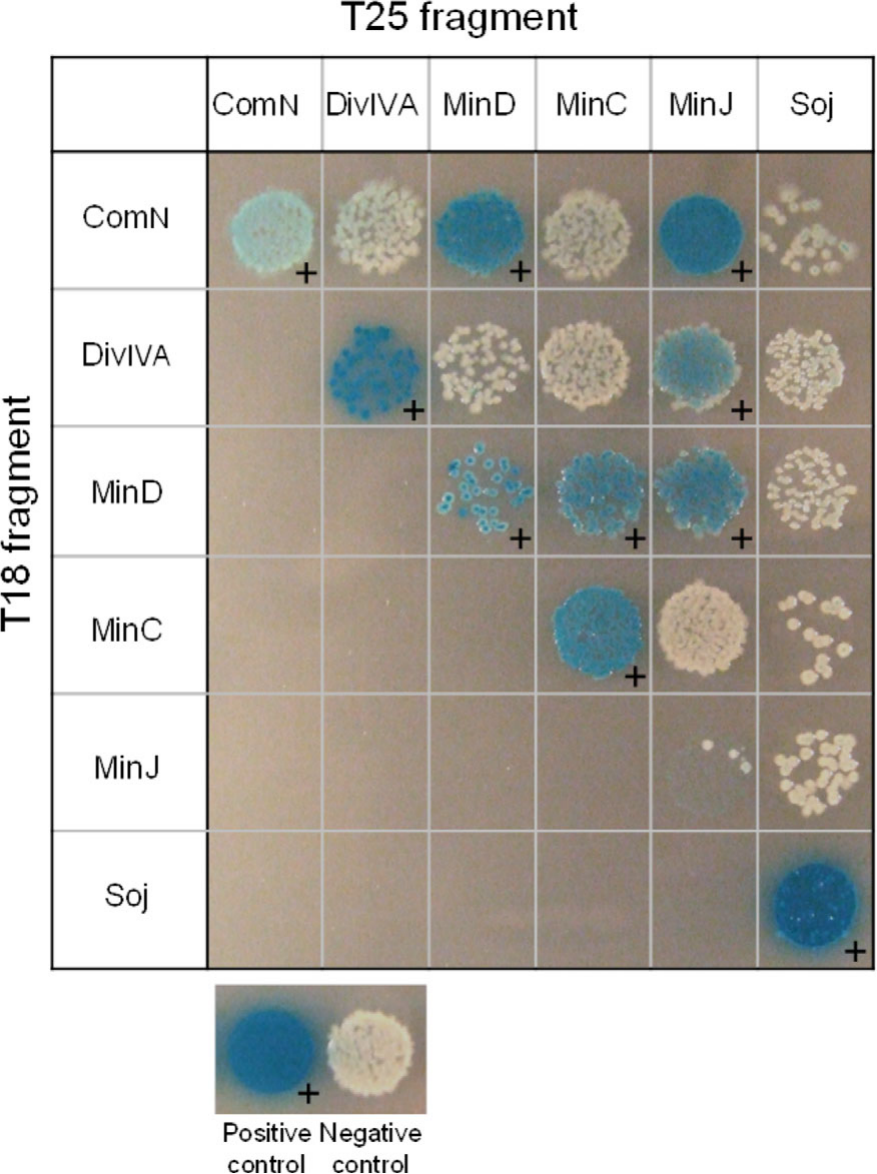
ComN interacts with both MinJ and MinD as assessed by bacterial two‐hybrid studies. Interactions between ComN, DivIVA, MinD, MinC, MinJ and Soj proteins were tested using bacterial two‐hybrid assays. Only one combination of the interactions is shown for simplicity. A plus sign (**+**) denotes a positive interaction. Labels on the left of the image correspond to the T18 fusions cloned into the pUT18 plasmid and labels on the top of the image correspond to the T25 fusions cloned into the pKT25 plasmid. ComN, DivIVA and Soj were N‐terminal fusions to T18 on pUT18, whereas MinD, MinC and MinJ were C‐terminal fusions to T18. MinC and MinJ are N‐terminal fusions to T25 on pKT25, whereas ComN, DivIVA, MinD and Soj are expressed as C‐terminal fusion to T25. The positive control is the two halves of a leucine zipper from GCN4 in yeast fused C‐terminal to T18 and T25 in pUT18 and pKT25 and the negative control is empty pUT18 and pKT25 plasmids (Karimova *et al*., 1998).

To identify which of the putative protein‐protein interactions predicted by the results described above might be direct, we carried out systematic bacterial two‐hybrid analyses (Karimova *et al*., 2005). The results, shown in Fig. 5, confirmed the previously detected self‐interactions of MinD, MinC, MinJ, DivIVA, Soj and ComN (Bramkamp *et al*., 2008; Scholefield *et al*., 2011; dos Santos *et al*., 2012), as well as interactions between DivIVA‐MinJ, MinJ‐MinD and MinD‐MinC (Bramkamp *et al*., 2008). Our tests did reveal new interactions between ComN and MinJ, and ComN and MinD (see also Supporting Information Fig. S7), consistent with these proteins acting as a complex. Our tests did not show an interaction between ComN and DivIVA as previously detected by yeast two‐hybrid (dos Santos *et al*., 2012), probably because the adenylate cyclase fusion constructs were not fully functional for the interaction.

We did not detect interactions between Soj and the other polar proteins even though a positive Soj self‐interaction was obtained. It is possible that fusion of Soj to fragments of adenylate cyclase interfered with its interaction with some of the partners but still allowed Soj self‐interaction. It has previously been reported that expression of Soj in *E. coli*, without Spo0J, is deleterious to the cells (Hester and Lutkenhaus, 2007). It will be interesting to test whether interactions with Soj could occur when Spo0J is co‐expressed in the system.

## Discussion

### Three new proteins involved in prespore chromosome segregation

Previous work established that capture of the *oriC* region at the pole of the cell was crucial for successful prespore chromosome segregation, and that this capture was probably dependent on two distinct mechanisms based on the Soj‐Spo0J and RacA systems (reviewed in Errington, 2010). RacA is present only in sporulating cells and it operates via a relatively simple, well‐defined mechanism involving binding to multiple specific DNA binding (*ram*) sites close to *oriC* and recruitment to the cell pole via a direct interaction with DivIVA (Ben‐Yehuda *et al*., 2003; Wu and Errington, 2003; Ben‐Yehuda *et al*., 2005; Lenarcic *et al*., 2009). The Soj‐Spo0J system has proved more difficult to characterize but seems also to be dependent on DivIVA, directly or indirectly (Wu and Errington, 2003; Perry and Edwards, 2004; Perry and Edwards, 2006). In this work we have identified three additional proteins specifically involved in the Soj‐Spo0J pathway.

MinJ probably works indirectly by linking MinD to DivIVA (Bramkamp *et al*., 2008; Patrick and Kearns, 2008; van Baarle and Bramkamp, 2010). The protein has 6 predicted transmembrane segments (van Baarle and Bramkamp, 2010) and a likely protein interaction (PDZ) domain, but its biochemical function, other than participating in complexes of proteins at the cell pole, remains unclear.

Although previous work has hinted at a role for MinD in the localization of Soj (Autret *et al*., 2001; Murray and Errington, 2008), the absence of MinD is not known to affect the function of Soj in regulating DnaA, and *minD* mutants do not have a replication over‐initiation phenotype that is associated with the deletion of *soj* (RL and LJW, unpublished). We were therefore surprised to find that MinD (but not MinC) was clearly involved in polar *oriC* placement. MinD has a very well characterized role in division site selection in which it recruits and activates the division inhibitor MinC. Localization of MinC at nascent septum and cell poles prevents the FtsZ‐based division machine from assembling away from its proper mid cell division site (Gregory *et al*., 2008; reviewed by Lutkenhaus, 2007; Rowlett and Margolin, 2015). Involvement of the MinD protein in correct placement of the *oriC* region could explain various old results including its influence on Soj localization (see below) and the curious observation of reverse SpoIIIE pumping (Sharp and Pogliano, 2002). SpoIIIE protein uses skewed octameric SRS sequences to orientate DNA pumping (Ptacin *et al*., 2008; Cattoni *et al*., 2013; Fiche *et al*., 2013). In wild‐type cells in which the *oriC* region is correctly captured in the prespore compartment, DNA translocation is directed from mother cell to prespore. However, if the *oriC* region lies outside the prespore, as we now show frequently occurs in *minD* mutants, translocation could be misdirected away from the prespore, leading to reverse transfer of the prespore chromosome. We predict that this should also occur in the various other mutants that fail to capture *oriC* in the prespore compartment.

**Figure 6:**
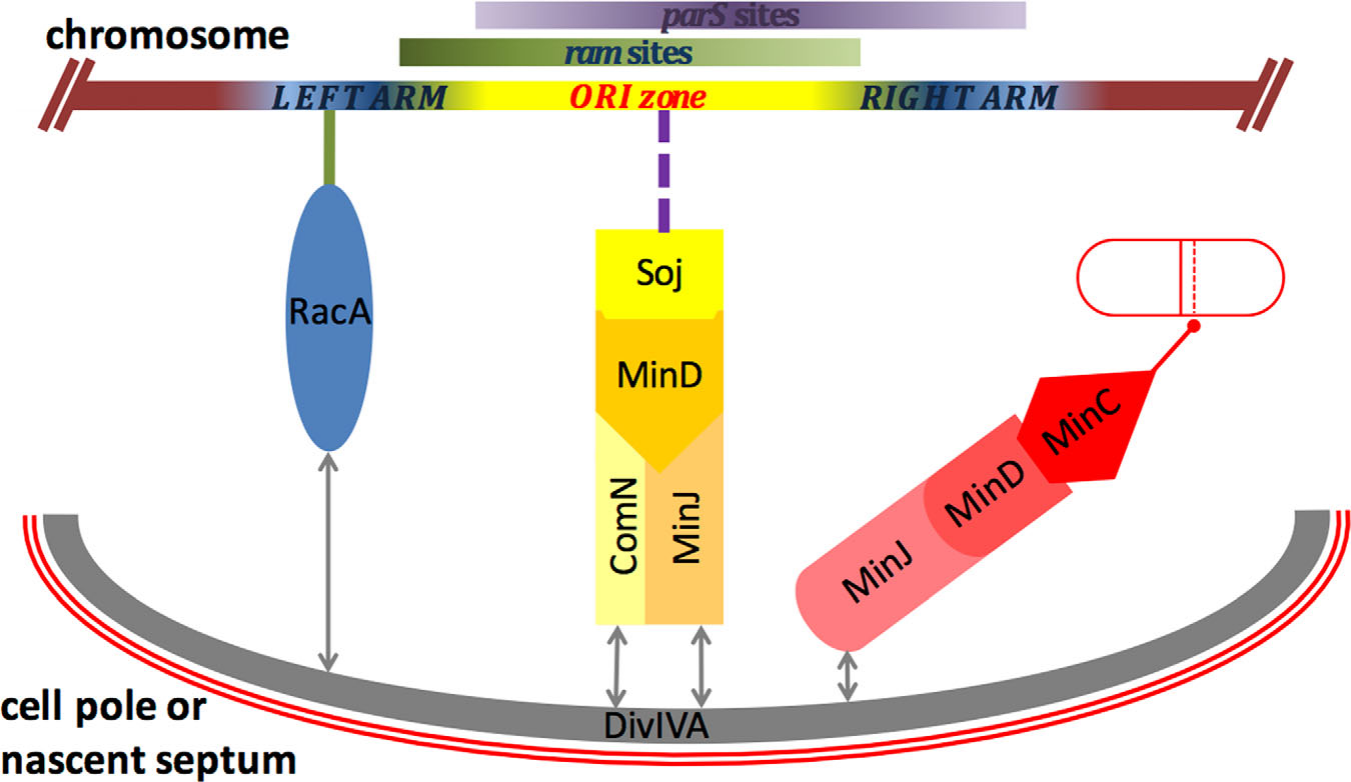
A model summarizing the interactions of the main players involved in placing the *oriC* region of the chromosome at the cell pole during spore development. For simplicity Spo0J is omitted in this model, though it is known that it binds directly to the *parS* sites on the chromosome and that it regulates the activity of Soj. Two systems are required to ensure that the whole of the *oriC* region (upper part) is correctly located at the pole to be included in the prespore upon asymmetric septation. Both are associated with the cell pole through a direct interaction with the DivIVA protein at the pole. The RacA system (in blue), present only during sporulation, particularly affects the ARM zone of the chromosome (solid green line) through directly binding to the *ram* sites located mainly to the left of *oriC* as indicated by the green bar. The other system (shades of yellow) consists of vegetative proteins MinD, MinJ, ComN and Soj, all located at the cell pole. MinD is recruited to DivIVA through MinJ. One population of MinD also interacts with ComN, which stabilizes the MinJ/MinD complex at the cell pole and also allows MinD to recruit/activate Soj, directly or indirectly. Soj probably acts on DNA (broken purple line), either at the *parS* region (purple area) through Spo0J (not shown), or through another protein to bring the ORI zone of the chromosome to the cell pole. It is also possible that Soj stimulates MinD to bind DNA directly (not shown). When not in complex with ComN, MinD carries out its Min function (outlined in red) by recruiting and activating the division inhibitor MinC, to block cell division near the poles and nascent septum (broken red line in the red cell on the right).

ComN was previously implicated in the regulation of competence (Ogura and Tanaka, 2009; dos Santos *et al*., 2012). This finding did not offer an obvious reason for localization of the protein at the cell pole via DivIVA, but its involvement in polar *oriC* capture now provides a functional explanation. The ComN protein is small and highly charged, and although its precise function in *oriC* positioning remains to be uncovered, our results suggest that ComN separates the two specific activities of MinD: ComN in complex with MinJ stabilizes and primes MinD to regulate *oriC* positioning in the cell, together with Soj, whereas the MinJ‐MinD complex allows proper control of division site selection *via* MinC (Fig. 6).

### A polar segregation complex targeted on Soj

Several lines of evidence point to the proteins mentioned above forming an interactive complex at the cell pole (summarized in Fig. 6), which contributes to *oriC* positioning at least partly by modulating the localization and or activity of Soj protein. First, all of the proteins have been visualized at the cell poles in sporulating and vegetative cells by imaging methods. Second, localization dependence experiments here and previously (Bramkamp *et al*., 2008; Murray and Errington, 2008; Patrick and Kearns, 2008; dos Santos *et al*., 2012) show that there is a hierarchy of dependence, with DivIVA as the primary determinant of polar localization, interacting with MinJ and ComN, MinD and finally Soj lying downstream (Fig. 6). Lastly, although other methods are needed to further identify and verify the interactions within the complex, bacterial two‐hybrid experiments have identified likely direct pairwise interactions between several of the proteins (Fig. 5).

Previous work on the Soj protein has revealed a complex and dynamic localization pattern involving binding to the nucleoid, Spo0J, DnaA and the division septum (Marston and Errington, 1999a; Quisel *et al*., 1999; Hester and Lutkenhaus, 2007; Murray and Errington, 2008). The different localization sites are thought to be dependent on different nucleotide associated states and or monomer/dimer forms (Murray and Errington, 2008). The septal localization was previously shown to depend on MinD protein but not MinC (Murray and Errington, 2008). Although we are still some way off understanding the precise role of the Soj protein in prespore chromosome capture, it now seems that the association of Soj with the septum is important for *oriC* capture during sporulation and that this activity is influenced by the polar segregation complex of proteins identified here. Three other lines of evidence support this idea. First, deletions of *minJ*, *comN* and *minD*, all generate an ‘ORI zone out’ chromosome defect, similar to that of the *soj* mutant, if not stronger (Fig. 2) (Sullivan *et al*., 2009). Second, mutation of either *comN* or *minD* exacerbates the effects of a *racA* mutation on prespore chromosome capture, just as described for *racA* and *soj* previously (Wu and Errington, 2003). Third, *soj*, *minD* and *comN* seem to work in the same pathway.

At present, based on analysis of the localization hierarchy and two‐hybrid results, MinD is the most likely candidate for the immediate target for Soj interaction at the cell pole (Fig. 6). Intriguingly, MinD is closely related to Soj in structure and mechanism of regulation. Both proteins are deviant Walker ATPase proteins. They both undergo reversible dimerization, which is regulated by ATP binding and hydrolysis (Lutkenhaus, 2012). It is therefore possible that the two proteins interact and, either become active for DNA binding and movement (e. g., by rebalancing the Soj cycle), or are able to stimulate the activity of another protein to bring the *oriC* to the pole. Recently it has been shown that *E. coli* MinD binds directly to DNA and plays a role in chromosome segregation (Di Ventura *et al*., 2013), raising the possibility that *B. subtilis* MinD could also act on DNA directly under certain conditions. A detailed understanding of the nature of these putative regulatory interactions will require further investigation, including biochemical characterization of the proteins involved.

The dual functionality of MinD in both *B. subtilis* and *E. coli* raises the possibility that MinD could also play an important role in chromosome segregation in other organisms. Interestingly, many species of Clostridium, a group of Gram‐positive, anaerobic endospore formers, lack a gene encoding RacA (http://string-db.org/). It is therefore possible that during sporulation chromosome segregation in these bacteria is carried out by the MinD/Soj/ComN pathway studied here.

### Comparison with other chromosome segregation systems

The *parABS* systems have been widely studied as factors implicated in the active segregation of bacterial chromosomes and plasmids. However, several aspects of their function remain poorly understood. Given their relative simplicity – two small proteins and a recognition site – they are surprisingly diverse in apparent function. Thus, as well as active *oriC* segregation, they have been implicated in recruitment of SMC complexes to origins, positive and negative regulation of DNA replication, transcriptional regulation, and other diverse processes (Marston and Errington, 1999a; Quisel *et al*., 1999; Gerdes *et al*., 2000; Livny *et al*., 2007; Murray and Errington, 2008; Gruber and Errington, 2009; Sullivan *et al*., 2009). The role of the *Caulobacter crescentus parABS* system in chromosome segregation is now well understood. In this Gram‐negative α‐proteobacterium, the *parABS* system clearly plays a central role in chromosome segregation. Initiation of DNA replication takes place with the parent origin located at the old cell pole. The ParB‐*parS* complex of one daughter origin then migrates rapidly to the new cell pole mediated by a ‘ParA relay’ mechanism (Lim *et al*., 2014) in which the *oriC*‐ParB‐*parS* complex migrates through a ‘cloud’ of DNA‐bound ParA molecules, stimulating ATP hydrolysis and dissociation of ParA dimers in the process (Ptacin *et al*., 2010; Schofield *et al*., 2010; Shebelut *et al*., 2010; Lim *et al*., 2014). Ultimately, the origin complex is captured at the new cell pole through interaction with two polarly localized proteins, PopZ (Bowman *et al*., 2008) and TipN (Ptacin *et al*., 2010; Schofield *et al*., 2010).

The relatively fast and directed movement of ParB(Spo0J)*‐parS* complexes has not been seen in vegetative growth of *B. subtilis*, neither has any role for the cell poles in vegetative chromosome segregation. Nevertheless, it is interesting that both *comN* and *minD*, but not *minC* mutants, have an increased *oriC*‐pole distance. We have previously shown that absence of MinD does not only affect the septal recruitment of Soj but also causes Spo0J foci to be less condensed compared to the Min^+^ cells. These observations raise the possibility that the MinJ/ComN/MinD polar complex also plays a role in chromosome segregation of vegetative *B. subtilis* cells.

In contrast to vegetative cells, the sporulation specific movement of *oriC* regions to the extreme cell poles may be equivalent to that of *C. crescentus*. Indeed, the sporulation segregation process now seems also to involve a series of polarly located proteins in *B. subtilis*, although there does not seem to be any clear primary sequence similarity between the polar proteins of the two organisms. In the light of the recent speculations about the possible evolutionary origins of the Gram‐negative bacteria from an ancestral endospore forming bacterium (Tocheva *et al*., 2011; Errington, 2013) the apparent similarity between α‐proteobacterial and endospore chromosome segregation mechanisms is highly intriguing.

## Experimental procedures

### General methods

Strains and primers used in this study are listed in Supporting Information Tables S1 and S2, respectively. General DNA manipulation in *E. coli* was done as described (Sambrook *et al*., 1989). Nutrient agar (Oxoid) was routinely used as a solid medium for growth of *E. coli* and *B. subtilis*. Liquid media used were Luria–Bertani (LB) or casein hydrolysate (CH) medium (See (Harwood and Cutting, 1990). Competence for genetic transformation was induced in *B. subtilis* as described previously (Hamoen *et al*., 2002). *B. subtilis* was induced to sporulate by the resuspension method [90], as modified by Partridge and Errington (Sterlini and Mandelstam, 1969). For blue/white selection 0.008% of X‐gal was used. Supplements were added as required: 100 μg/ml Ampicillin, 20 μg/ml tryptophan, 5 μg/ml chloramphenicol, 2 μg/ml (for all *spoIIIE36‐kan* strains, and for all alleles of the *soj‐spo0J* locus containing the *neo* gene) or 5 μg/ml kanamycin (all other strains containing a *kan* marker, notably mutants obtained with the random mutagenesis screens using pMarB), 50 μg/ml spectinomycin, 5 μg/ml zeocin, 1 μg/ml phleomycin, 1 μg/ml erythromycin and 25 μg/ml lincomycin. Random transposon mutagenesis was performed using pMarB as described (Le Breton *et al*., 2006). Colonies were grown for up to 36 h to select blue ones by visual inspection.

### Construction of strains and plasmids

To construct a *lacZ* driven by the *spoIIQ* promoter (*P_spoIIQ_*) containing a *lacO* operator for artificial repression by LacI, a DNA fragment containing the P_*spoIIQ*_ was PCR amplified with primer pair oTK20/oTK21 from chromosomal DNA of *B. subtilis* and cloned as an *Hin*dIII/*Bam*HI fragment in pMUTIN4, which was amplified with primer pair oTK22/oTK23, using plasmid pMUTIN4 ((Vagner *et al*., 1998) as a template. In this way the *spac* promoter was removed and replaced by the *B. subtilis spoIIQ* promoter (without its RBS), upstream of the *lacO* operator (*lacO‐oid*, AATTGTGAGCGCTCACAATT, see (Vagner *et al*., 1998). The resulting plasmid, pTK31, was used as a template to PCR amplify the *P_spoIIQ_‐lacO*(oid)‐*lacZ*, together with the terminators located upstream (*rrnB1* + *rrnB2* from the original pMUTIN4 plasmid) and erythromycin gene using primer pair oTK24/oTK25. This fragment was subsequently ligated to fragments (1.5–2.0 kb) of chromosomal DNA of *B. subtilis* flanking the desired integrations sites, PCR amplified with primer pairs oTK26/oTK27 and oTK28/oTK29 [integration at *amyE*, (+327 kbp)], oTK34/oTK35 and oTK36/oTK37 [integration next to *jag* (−3 kbp)] or oTK30/oTK31 + oTK32/oTK33 (integration next to *spoIID*, (−438 kbp). Ligation mixtures were then transformed into *B. subtilis* wild‐type strain 168CA and transformants with correct integration of the *erm*‐terminator‐*P_spoIIQ_‐lacO‐lacZ* construct were selected and verified by phenotypic analyses.

A construct containing the *spec‐P_spoIIQ_‐lacI* cassette was made by fusing a DNA fragment containing P*_spoIIQ_*, PCR amplified from chromosomal DNA of *B. subtilis* using primers oTK48/oTK49 (*P_spoIIQ_*), a PCR product amplified from plasmid pMUTIN4 using primers oTK50/oTK51 (*lacI*, with a *lambda0* terminator downstream to prevent inward reading), and the spectinomycin resistance gene (*spec*) PCR amplified from plasmid pAPNC213 (Morimoto *et al*., 2002) using primers oTK77/oTK78. The fused PCR product was digested with *Eco*RI and *Hin*dIII and cloned into plasmid pBS‐KS^‐^ (Stratagene) digested with the same restriction enzymes, yielding plasmid pTK32. Next, the entire *spec‐P_spoIIQ_‐lacI* cassette was amplified from plasmid pTK32, using primers oTK46/oTK47 and ligated to the DNA fragments flanking the intergenic region between *yxiF* and *yxxG* (−192 kbp), amplified with primer pairs oTK42/oTK43 and oTK44/oTK45. This ligation mixture was then directly transformed into the wild‐type strain 168CA and colonies containing the correct integration of the entire *spec‐P_spoIIQ_‐lacI* construct were verified using PCR.

The *spoIIIE36* mutation [which we here also denote as *spoIIIE*(frozen)] containing the G1285=>A transition in the *spoIIIE* ORF, originally isolated by Piggot (1973; Wu and Errington, 1994) was introduced into the chromosome of *B. subtilis* wild‐type strain 168CA by fusing PCR products of the *spoIIIE* gene generated from chromosomal DNA of the *spoIIIE36* mutant strain 36.3 with primer pairs oTK53/oTK54, oTK55/oTK56 and oTK59/oTK61, and a PCR product containing the kanamycin resistance gene was PCR amplified using oTK57/oTK58 from plasmid pHT21 (Trieu‐Cuot and Courvalin, 1983), in a single reaction by means of overlap‐extension PCR. The resulting PCR mixture was directly transformed into the wild‐type strain 168CA, and transformants were selected on plates containing 2 μg/ml kanamycin. Clones containing both the *spoIIIE36* mutation and the *kan* gene downstream of *spoIIIE36*, were verified by phenotypic characterization (Wu *et al*., 1995). Insertion of a *cat* gene downstream of the *spoIIIE36* allele, replacing the *kan* gene, was done by ligating PCR products generated using primer pairs oTK53/oTK66 and oTK67/oTK68 from the chromosomal DNA of the *spoIIIE36*‐*kan* strain, and oTK64/oTK65 from plasmid pSG1186 (for the *cat* gene), which were digested with the relevant restriction enzymes. The ligation mixture was directly transformed into the wild‐type strain 168CA and Cam^R^, Kan^S^, Spo^‐^ colonies were selected and verified by sequencing. In the same way, a *zeo* gene was placed downstream of *spoIIIE36*, amplified using primers oTK69/oTK70 from plasmid p7Z6 [97].

A marker‐less *comN* deletion mutant was constructed using the *cre‐lox* system as described (Yan *et al*., 2008), using PCR products generated with primer pairs oTK71/oTK72, oTK73/oTK74 from chromosomal DNA of *the* wild‐type strain 168CA, and oTK75/oTK76 from plasmid p7Z6. A 47 bp fragment of the *comN* ORF and the scar of the *cre‐lox* system were left behind encoding the following peptide: VEKHPADPTVRIAYIIRTVGSQEALEELLR.

A *minD* deletion mutant (marker‐free) was constructed by growing strain *ΔminD::spec P_spac_‐mazF* two consecutive times on NA plates containing 1 mM IPTG and selecting for spectinomycin‐sensitive clones.

A P*_comN_‐comN‐gfp* fusion was made by cloning a PCR product encompassing P*_comN_‐comN*, which was generated from *B. subtilis* 168CA chromosomal DNA with primers oTK10/oTK11 into the *Sph*I, *Kpn*I digested pSG1154mgfp [a monomeric GFP variant made by Henrik Strahl based on pSG1154 of (Lewis and Marston, 1999)], thereby removing *P_xyl_*. This construct, pTK11, was transformed into the relevant *B. subtilis* strains and *spec^R^*, *amyE* ^‐^ transformants were selected and used for further study.

An IPTG‐inducible *mcherry‐comN* fusion was made by cloning a PCR product, generated on chromosomal DNA of *B. subtilis* 168CA with primers oTK7/oTK9, into the *BamH*I, *EcoR*I sites of plasmid pAPNC(*cat*)‐*mcherry* (Henrik Strahl). The resulting construct was integrated into the *aprE* locus of the relevant strains of *B. subtilis*, selecting for chloramphenicol resistant colonies.

A P*_comN_‐comN* construct for complementation of the *comN* deletion was made by cloning a PCR product encompassing P*_comN_‐comN*, which was generated from *B. subtilis* 168CA chromosomal DNA with primer pair oTK10/oTK14 into *Sph*I, *Not*I digested pSG1154mGFP, thereby removing both P_*xyl*_ and *gfp*. This construct, pTK13, was transformed into the relevant *B. subtilis* strains and *spec^R^*, *amyE* ^‐^ transformants were selected and used for further experiments.

Strains containing the *P_spoIIQ_‐yfp* and *P_spoIIQ_‐cfp* reporters were constructed by transforming the relevant plasmids described in (Sullivan *et al*., 2009) into *B. subtilis*, selecting for the appropriate antibiotic resistance and checking for double cross‐over recombination.

### Microscopy

To visualize cells overnight cultures grown at 30°C in CH medium were diluted 1:100 into fresh medium. For sporulation, cells were grown at 37°C. For visualisation of protein fusions to GFP, cells were grown at 30°C. 0.7 µl cells were spotted onto thin ∼1.2% agar pads (containing 10% of the growth medium). Microscopic visualisation of cells was carried out as described (Murray and Koh, 2014; Adams *et al*., 2015). Membranes were stained with 0.4 µg.ml^−1^ FM5‐95 dye (Invitrogen). When necessary data were analysed using the Metamorph software (fluorescent trapping assays) and ImageJ (http://rsb.info.nih.gov/ij) containing the ObjectJ plugin (cell length and *oriC*‐pole distance measurements). Standard deviations are from at least three experiments.

Two colour colocalisation imaging was performed using a Nikon Eclipse Ti microscope equipped with a Nikon CFP APO TIRF x100/1.49 oil objective, 488 nm and 561 nm solid‐state lasers, and Andor Xion X3 EMCCD camera. All image capture was conducted using Nikon NIS elements 4.0. Cells were immobilized on 1.5% agarose slides.

### Bacterial two‐hybrid interaction tests

For bacterial two‐hybrid assays, *comN* was PCR amplified from chromosomal DNA of *B. subtilis* wild‐type strain 168CA using primer pairs oTK11/oTK15, and cloned into the *Kpn*I, *Xba*I sites of plasmids pKT25 and pUT18 (Karimova *et al*., 2005) to create both N‐ and C‐terminal fusions to adenylate cyclase fragments T25 and T18. Other plasmids for bacterial‐two‐hybrid analysis were obtained from the Daniel Strain Collection, Newcastle University. The experiment was carried out as previously described (Daniel *et al*., 2006). 10 µl of the cotransformation mixture was plated onto plates containing X‐gal (0.004%). Plates were incubated at 30°C for 48 h, followed by room temperature for 24 h.

## Supporting information

Supporting Information
