# Supplementary material for: Complex polar machinery required for proper chromosome segregation in vegetative and sporulating cells of Bacillus subtilis
Source: Mol Microbiol. 2016 May 18;101(2):333–50. doi: 10.1111/mmi.13393 (PMC4949633; doi:10.1111/mmi.13393)
Supplement: Supplementary file 1 — Supporting Information [file MMI-101-333-s001.pdf]

Supplementary Figures and Tables Kloosterman TG et al, 2016

Figure S1: Illustration of the blue-white screen for mutants with an 'ORI zone out' phenotype. An otherwise wild-type strain with the *spoIIIE*(frozen) mutation carrying P_{spoIIQ}-*lacI* at the ORI zone (at *yxiF*, -192 kbp) and P_{spoIIQ}-*lacO-lacZ* at the ARM zone (at *amyE*, +327 kbp) grown for 20 hours at 37°C on Nutrien Agar plates to allow for sporulation, in the absence (top panel) or presence (bottom panel) of 1 mM IPTG.

Figure S2: Complementation of the *comN* in-frame deletion mutant. All strains contained *spoIIIE*(frozen)-*cat* and *jag*::P_{spoIIQ}-*lacO -lacZ-erm* (*oriC* proximal; -3 kbp), in addition to the genotypes indicated in the figure. Cells were grown for 20 hours on NA plates supplemented with X-gal. Empty vector, pSG1154. Of note, in this figure the *lacZ* was at the ORI zone and the *lacI* construct used in the screen was NOT present, explaining the blue color of the wild-type and the whiter color of the *comN* mutant.

Figure S3: Use of the P_{spoIIQ}-based fluorescent reporter assay to quantify trapping of ORI and ARM zones in the prespore compartment of the wild-type and the *comN* mutant as described in Fig 2A. The ORI zone is represented by a P_{spoIIQ}-*yfp* construct inserted at *yycR* (-79 kbp) and the ARM zone is represented by a P_{spoIIQ}-*cfp* construct inserted at *amyE* (+327 kbp). The *comN* mutant used here was a transposon mutant *comN::Tn-kan* isolated from the transposon screen, and the wild-type strain was a random white but kanamycin-resistant colony from the transposon screen.

Figure S4: Direct localization of *oriC* in sporulating cells 3 hours after the induction of sporulation, using a *tetO* array at *yycR* (-79 kbp) visualised by binding of TetR-GFP [66]. **A.** Epifluorescent microscopic images showing localization of *oriC* inside the wild-type prespore (arrow in the top panel) and mislocalized *oriCs* in the *comN* mutant cells (arrows in the bottom panel). Membranes were stained with FM5-95. Cells contained a wild-type copy of *spoIIIE*. **B.** Quantification of localization of *oriCs* in- or outside the prespore compartment in wild-type, and in *comN* and *minD* mutants, by directly visualizing *oriC* using the TetR-GFP/*yycR*::$tetO_{25}$ construct. Strains contained the *spoIIIE*(frozen) mutation. **C.** Same as in **B**, but now in the *spoIIIE* wild-type ($spoIIIE^{+}$) background. At least 300 cells per strain were counted. Data show a representative experiment.

Figure S5: A. Cell length distribution of selected wild-type and *comN* mutant cells (from the data set in Fig. 3A) that have the same cell length. Cells were grown in sporulation conditions. On the basis of these collections of cells, *oriC*-pole distances were determined for Fig. S5B.

B. *OriC*-pole distances determined as described in Fig. 3, based on a selection of cells from (A) that had the same length distributions in the two strains (see Fig. S5A). **C.** *comN* and *minD* mutant strains have increased cell length compared to the wild-type strain. At least 350 cells were measured/counted.

Figure S6: Epifluoresence images showing the localization of ComN-GFP in the wild-type and the *minD*, *minC*, *minJ* and *divIVA* mutants. Cells were grown to mid-logaritmic phase in CH medium and ComN-GFP was expressed from P_{comN}-*comN-gfp-spec* at *amyE* .

Figure S7: Results of all bacterial-two-hybrid plasmid combinations for the newly identified ComN-MinD and ComN-MinJ interactions. Genes were cloned into pUT18 and pKT25 plasmids both N-terminal and C-terminal to the adenylate cyclase fragments and each combination of these plasmids is displayed in the figure, where positive interactions are indicated by a plus (**+**) sign. The -N and -C after the plasmid denote whether the gene is cloned N or C-terminal to the adenylate cyclase fragment. The positive control is the two halves of a leucine zipper from GCN4 in yeast fused C-terminal to T18 and T25 in pUT18 and pKT25 and the negative control is empty pUT18 and pKT25 plasmids, as in Figure 5 (Karimova et al, 1998).

Table S1. Strains used in this study

strain	parent	*genotype*	source
168ed	ED	*trpC2*	(Kobayashi *et al.*, 2003)
168ca	CA	*trpC2*	(Kunst & al., 1997)
TK43	CA	*spoIIIE36-cat jag::*P_{IIQ}*-lacO-oid-lacZ; erm*	This study
TK52	CA	*ΔminD spoIIIE36-cat jag::*P_{IIQ}*-lacO-oid-lacZ; erm*	This study
TK53	CA	*ΔminD spoIIIE36-cat jag::*P_{IIQ}*-lacO-oid-lacZ; erm ΔlacA::tet*	This study
TK506	CA	*ΔcomN spoIIIE36-cat jag::*P_{IIQ}*-lacO-oid-lacZ; erm*	This study
TK507	CA	*ΔcomN spoIIIE36-cat jag::*P_{IIQ}*-lacO-oid-lacZ; erm ΔlacA::tet*	This study
TK508	CA	*ΔminD Δsoj::neo spoIIIE36-cat jag::*P_{IIQ}*-lacO-oid-lacZ; erm*	This study
TK509	CA	*ΔcomN Δsoj::neo spoIIIE36-cat jag::*P_{IIQ}*-lacO-oid-lacZ; erm*	This study
TK309	CA	*ΔcomN spoIIIE36-cat jag::*P_{IIQ}*-lacO-oid-lacZ; erm amyE::PcomN-comN-gfp; spec*	This study
TK281	CA	*ΔcomN spoIIIE36-cat jag::*P_{IIQ}*-lacO-oid-lacZ; erm amyE::PomN-comN; spec*	This study
TK282	CA	*ΔcomN spoIIIE36-cat jag::*P_{IIQ}*-lacO-oid-lacZ; erm amyE::pSG1154 integration; spec*	This study
TK57	CA	*spoIIIE36-cat yxiF::*P_{IIQ}*-lacI; spec amyE::*P_{IIQ}*-lacO-oid-lacZ; erm ΔlacA::tet*	This study
TK72	CA	*spoIIIE36-cat yxiF::*P_{IIQ}*-lacI; spec spoIID::*P_{IIQ}*-lacO-oid-lacZ; erm ΔlacA::tet*	This study
TK501	CA	*ΔminD spoIIIE36-cat yxiF::*P_{IIQ}*-lacI; spec amyE::*P_{IIQ}*-lacO-oid-lacZ; erm*	This study
TK502	CA	*Δsoj::neo spoIIIE36-cat yxiF::*P_{IIQ}*-lacI; spec amyE::*P_{IIQ}*-lacO-oid-lacZ; erm*	This study
TK75	CA	*spoIIIE36-cat yycR::*P_{IIQ}*-yfp (-7°); spec ywjI::*P_{IIQ}*-cfp (-35°); erm*	This study
TK96	CA	*ΔcomN spoIIIE36-cat yycR::*P_{IIQ}*-yfp (-7°); spec ywjI::*P_{IIQ}*-cfp (-35°); erm*	This study
TK156	CA	*ΔminD spoIIIE36-cat yycR::*P_{IIQ}*-yfp (-7°); spec ywjI::*P_{IIQ}*-cfp (-35°); erm*	This study

TK164	CA	*Δsoj::neo spoIIIE36-cat yycR::P_{IIQ}-yfp (-7°); spec ywjI::P_{IIQ}-cfp (-35°); erm*	This study
TK190	CA	*ΔcomN::zeo ΔminD spoIIIE36-cat yycR::P_{IIQ}-yfp (-7°); spec ywjI::P_{IIQ}-cfp (-35°); erm*	This study
TK167	CA	*ΔcomN Δsoj::neo spoIIIE36-cat yycR::P_{IIQ}-yfp (-7°); spec ywjI::P_{IIQ}-cfp (-35°); erm*	This study
TK198	CA	*ΔminD Δsoj::neo spoIIIE36-cat yycR::P_{IIQ}-yfp (-7°); spec ywjI::P_{IIQ}-cfp (-35°); erm*	This study
TK183	CA	*ΔminC::kan spoIIIE36-cat yycR::P_{IIQ}-yfp (-7°); spec ywjI::P_{IIQ}-cfp (-35°); erm*	This study
TK248	CA	*ΔminC::kan ΔminJ::tet spoIIIE36-cat yycR::P_{IIQ}-yfp (-7°); spec ywjI::P_{IIQ}-cfp (-35°); erm*	This study
TK205	CA	*ΔracA::tet spoIIIE36-cat yycR::P_{IIQ}-yfp (-7°); spec ywjI::P_{IIQ}-cfp (-35°); erm*	This study
TK206	CA	*ΔcomN ΔracA::tet spoIIIE36-cat yycR::P_{IIQ}-yfp (-7°); spec ywjI::P_{IIQ}-cfp (-35°); erm*	This study
TK81	ED	*comN::Tn(kan) spoIIIE36 yycR::P_{IIQ}-yfp (-7°); phleo amyE::P_{IIQ}-cfp (+28°); cat*	This study
TK82	ED	*neg control::Tn(kan) (clone 3.16) spoIIIE36 yycR::P_{IIQ}-yfp (-7°); phleo amyE::P_{IIQ}-cfp (+28°); cat*	This study
AK47	CA	*yycR::erm(tetO~25) amyE::spec(Pspac(c)-tetR-gfp)*	(Murray & Koh, 2014)
TK132	CA	*spoIIIE36-cat yycR::erm(tetO~25) amyE::spec(Pspac(c)-tetR-gfp)*	This study
TK127	CA	*ΔminD yycR::erm(tetO~25) amyE::spec(Pspac(c)-tetR-gfp)*	This study
TK130	CA	*ΔcomN yycR::erm(tetO~25) amyE::spec(Pspac(c)-tetR-gfp)*	This study
TK187	CA	*ΔminC::kan yycR::erm(tetO~25) amyE::spec(Pspac(c)-tetR-gfp)*	This study
TK159	CA	*ΔcomN Δsoj::neo yycR::erm(tetO~25) amyE::spec(Pspac(c)-tetR-gfp)*	This study
TK131	CA	*Δsoj::neo yycR::erm(tetO~25) amyE::spec(Pspac(c)-tetR-gfp) (HM766)*	This study
TK133	CA	*ΔcomN spoIIIE36-cat yycR::erm(tetO~25) amyE::spec(Pspac(c)-tetR-gfp)*	This study
TK134	CA	*ΔminD spoIIIE36-cat yycR::erm(tetO~25) amyE::spec(Pspac(c)-tetR-gfp)*	This study
TK283	CA	*amyE::P_{comN}-comN; spec*	This study
TK303	CA	*amyE::P_{comN}-comN-mgfp;spec*	This study
TK312	CA	*ΔminC::kan amyE::P_{comN}-comN-gfp;spec*	This study
TK313	CA	*ΔminD::erm amyE::P_{comN}-comN-gfp;spec*	This study
TK315	CA	*ΔdivIVA::tet amyE::P_{comN}-comN-gfp;spec*	This study
TK316	CA	*ΔminJ::tet amyE::P_{comN}-comN-gfp;spec*	This study
TK93	CA	*aprE::Pspac-mCherry-comN; cat*	This study
DMR111	CA	*aprE::Pspac-mCherry-comN; cat, minD::gfp-minD::kan*	This study
TK36	CA	*ΔminD::spec P_{spac}-mazF*	strain collection L Hamoen, unpublished
TK38	CA	*ΔminD*	This study
TK91	CA	*ΔcomN::zeo (cre/lox sites)*	This study
TK92	CA	*ΔcomN*	This study
TK144	CA	*ΔminC::kan*	L Hamoen unpublished
TK146	CA	*ΔminJ::tet*	(Bramkamp *et al.*, 2008)
TK110	CA	*ΔracA::tet*	(Wu & Errington, 2003)
TK105	CA	*ΔracA::erm*	laboratory stock
HM748	CA	*Δsoj::neo*	H Murray unpublished
SG82	-	*ΔlacA::tet*	laboratory stock
TK142	CA	*ΔcomN Δnoc::tet*	This study
TK147	CA	*ΔminD Δnoc::tet*	This study
DMR114	CA	*ΔminD:erm*	This study; (Marston et al., 1998)
DMR124	CA	*ΔracA::erm ΔcomN::zeo*	This study
EBS499	PY79	*minC4-gfp, sacA::tet*	(Gregory *et al.*, 2008)
JAG118	PY79	*minD4-gfp, sacA::P_{spoIIR}-creΩspec, amyE::P_{minCD}-minCDΩcat*	(Gregory et al., 2008)

TK288	PY79	*EBS499 ΔcomN::zeo*	This study
TK289	PY79	*JAG118 ΔcomN::zeo*	This study
TK152	CA	*ΔcomN minD::gfp-minD::kan*	This study
TK153	CA	*minD::gfp-minD::kan*	J. Schneeweiss/LJ Wu, unpublished
HM740	CA	*gfp-soj::neo*	H Murray unpublished
TK229	CA	*ΔcomN gfp-soj::neo*	This study
TK117	CA	*ΔminD gfp-soj::neo*	This study
HM70	ED	*gfp-soj::neo ΔminC::erm (in-frame deletion)*	(Murray & Errington, 2008)
TK305	ED	*gfp-soj::neo ΔminC::erm (in-frame deletion) ΔminJ::tet*	This study

Tn, insertion of mariner (*kan*) transposon from pMarB. Resistance gene abbreviations: cat, chloramphenicol; erm, erythromycin; kan, kanamycin; spec, spectinomycin; tet, tetracycline; zeo, zeocin; phleo, phleomycin; neo, neomycin.

Table S2. Primers used in this study.

primer name	sequence
OTK7	CGGGATCCGTGGAGAAGCATCCAGCC
OTK9	GCGAATTCAAACCTGTGCGAATCAAC
OTK10	GACATTTGCATGC CGATAAGGATAAGAGTGCCC
OTK11	GGGGGTACCTCTCAGCAGTTCTTCCAATG
OTK14	TAAGCGGCCGCATGTATATCCTCCCTTAAAC
OTK15	ACTTCTAGAGTGGAGAAGCATCCAGCCG
OTK20	CGGGATCCAATTGTGAGCGCTCACAATTCTCAGCAACATTCTGAACAC
OTK21	CCCCAAGCTTCGTTTTTGGCACTCCTCTC
OTK22	CCGGGCGGCCGCCGCGG
OTK23	CCCCAAGCTTCCCCGATCTTCATCACC
OTK24	GCGCACTAGTCTTGAAATAGTACATAATGG
OTK25	GCGCGCTAGCCGAAATGATACACCAATCAGTG
OTK26	GCGCACTAGTGAAAAGTTTTGTCTGATTTATG
OTK27	GAATTCATTGCTCGGGC
OTK28	CAATCGGAACACCAAAACC
OTK29	GCGCGCTAGCTCTTGCAAAAGTTTGTGAAG
OTK30	GCGCGCTAGCCTTAGATAAAAGATATTCAG
OTK31	AGTTGCAGCGACTGATTTGCG
OTK32	GCGCACTAGTAGTAGCTGAAACGGTTTAGAG
OTK33	TCCTTTGGAAGAAGTTCGGC
OTK34	GCGCACTAGTCATAAAACCGAAGTCCGAT
OTK35	AATCTCCAATGATTTCCCC
OTK36	GCGCGCTAGCCTATCTTTTATGGGAAATAAC
OTK37	GTAAAGATTAATTATAGGAGG
OTK42	ACTGTTTCAGCTTCTATAGC
OTK43	CGGGATCCCATTAAATAAGAGATGTTACC
OTK44	CGGAATTCTTCCATCAATAAATGCCCC

OTK45	GCGAAGACAAGCCATTCGC
OTK46	ACGACTCACTATAGGGCG
OTK47	AAGGGAACAAAAGCTGGG
OTK48	CGTTTTTGGCACTCCTCTC
OTK49	CAAAATCGTCTCCCTCCCTCAGCAACATTCTGAACACTTTTC
OTK50	GGAGGGAGACGATTTTGATG
OTK51	CGGAATTCTCAGAACGCTCGGTTGCCGCCGGGCGTTTTTTATGCGTTGCGCTCACTGCCCGC
OTK53	TAGGTTTATTGGTGTTAGG
OTK54	TTCAATTTAGACTCCAGCATTTCTTTCAGCGAAACCATTG
OTK55	ATGCTGGAGTCTAAATTGAATG
OTK56	GAAAGCGGAACTCCCTTC
OTK57	*GAAGGGAGTTCCGCTTTC*CAACTTTCAAAACAACTTTG
OTK58	CAGAGTATGGACAGTTGCGG
OTK59	GCAACTGTCCATACTCTGTATAGTTGTCAATAATG
OTK61	GGAGTTCCCGAGTGTCATC
OTK64	CGGGATCCGCTTTTTCGTACCTATTAATG
OTK65	CGCTCTAGAATTCTTCAACTAAAGCACCC
OTK66	CGCTCTAGAGAAAGCGGAACTCCCTTC
OTK67	CGGGATCCTATAGTTGTCAATAATG
OTK68	GCATCTAAAGCAGGAAGAGCC
OTK69	CGCTCTAGACATTATACGAAGTTATCTTG
OTK70	CGGGATCCGCTATACGAAGTTATTCAGTCC
OTK71	TAACAGCGAAGCTGAGCG
OTK72	ATCGGATCCGCTGGATGCTTCTCCACGAA
OTK73	ATCGGATCCCAGGAAGCATTGGAAGAACTG
OTK74	ACAGTATCAGGGTCCATACC
OTK75	ATCGGATCCTACCGTTCGTATAATGTATGCTATACGAAGTTATTCAGTCC
OTK76	ATCGGATCCTACCGTTCGTATAGCATACATTATACGAAGTTATCTTGATATGGC
OTK77	GAGAGGAGTGCCAAAAACGCAAATTGTGAAAGGATGTAC
OTK78	CTCAAGCTTCACTAAATTAAAG

Table S3. Sporulation efficiency of the indicated strains. Both the *comN* and the *minD* mutants show premature cell lysis during sporulation, which would result in over-estimation of sporulation efficiency for these mutants if determined by the conventional method of doing heat-resistant spore and total viable cell counts. As an alternative method, we used microscopy to count the number of the phase bright spores as a percentage of the total number of cells. At least 500 cells were counted per strain/condition.

**Strain**	**Sporulation efficiency at T_{5h} (%)**	**Sporulation efficiency at T_{6h} (%)**
168CA	27.5	73.7
comN	17.7	74.5
minD	19.1	64.0
minC	23.7	69.4
racA	18.4	53.8
racA comN	8.7	8.1

REFERENCES

Bramkamp, M., R. Emmins, L. Weston, C. Donovan, R. A. Daniel & J. Errington, (2008) A novel component of the division-site selection system of *Bacillus subtilis* and a new mode of action for the division inhibitor MinCD. *Mol. Microbiol.* 70: 1556-1569.

Gregory, J. A., E. C. Becker & K. Pogliano, (2008) *Bacillus subtilis* MinC destabilizes FtsZ-rings at new cell poles and contributes to the timing of cell division. *Genes Dev.* 22: 3475-3488.

Kobayashi, K., S. D. Ehrlich, A. Albertini, G. Amati, K. K. Andersen, M. Arnaud, K. Asai, S. Ashikaga, S. Aymerich, P. Bessieres, F. Boland, S. C. Brignell, S. Bron, K. Bunai, J. Chapuis, L. C. Christiansen, A. Danchin, M. Debarbouille, E. Dervyn, E. Deuerling, K. Devine, S. K. Devine, O. Dreesen, J. Errington, S. Fillinger, S. J. Foster, Y. Fujita, A. Galizzi, R. Gardan, C. Eschevins, T. Fukushima, K. Haga, C. R. Harwood, M. Hecker, D. Hosoya, M. F. Hullo, H. Kakeshita, D. Karamata, Y. Kasahara, F. Kawamura, K. Koga, P. Koski, R. Kuwana, D. Imamura, M. Ishimaru, S. Ishikawa, I. Ishio, D. Le Coq, A. Masson, C. Mauel, R. Meima, R. P. Mellado, A. Moir, S. Moriya, E. Nagakawa, H. Nanamiya, S. Nakai, P. Nygaard, M. Ogura, T. Ohanan, M. O'Reilly, M. O'Rourke, Z. Pragai, H. M. Pooley, G. Rapoport, J. P. Rawlins, L. A. Rivas, C. Rivolta, A. Sadaie, Y. Sadaie, M. Sarvas, T. Sato, H. H. Saxild, E. Scanlan, W. Schumann, J. F. Seegers, J. Sekiguchi, A. Sekowska, S. J. Seror, M. Simon, P. Stragier, R. Studer, H. Takamatsu, T. Tanaka, M. Takeuchi, H. B. Thomaides, V. Vagner, J. M. van Dijl, K. Watabe, A. Wipat, H. Yamamoto, M. Yamamoto, Y. Yamamoto, K. Yamane, K. Yata, K. Yoshida, H. Yoshikawa, U. Zuber & N. Ogasawara, (2003) Essential *Bacillus subtilis* genes. *Proc. Natl. Acad. Sci. U. S. A.* 100: 4678-4683.

Kunst, F. & e. al., (1997) The complete genome sequence of the Gram positive bacterium *Bacillus subtilis*. *Nature* 390: 249-256.

Marston, A. L., H. B. Thomaides, D. H. Edwards, M. E. Sharpe & J. Errington, (1998) Polar localization of the MinD protein of *Bacillus subtilis* and its role in selection of the mid-cell division site. *Genes Dev.* 12: 3419-3430.

Murray, H. & J. Errington, (2008) Dynamic control of the DNA replication initiation protein DnaA by Soj/ParA. *Cell* 135: 74-84.

Murray, H. & A. Koh, (2014) Multiple regulatory systems coordinate DNA replication with cell growth in Bacillus subtilis. *PLoS Genet.* 10: e1004731.

Wu, L. J. & J. Errington, (2003) RacA and the Soj-Spo0J system combine to effect polar chromosome segregation in sporulating *Bacillus subtilis*. *Mol. Microbiol.* 49: 1463-1475.

FIGURE S1

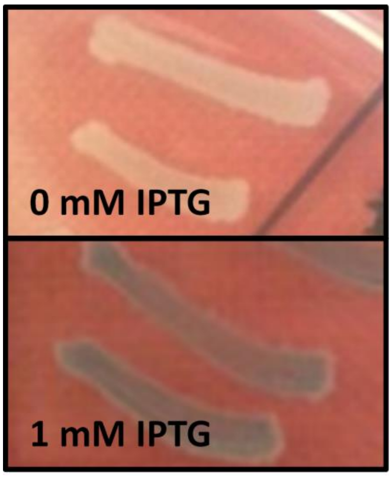

FIGURE S2

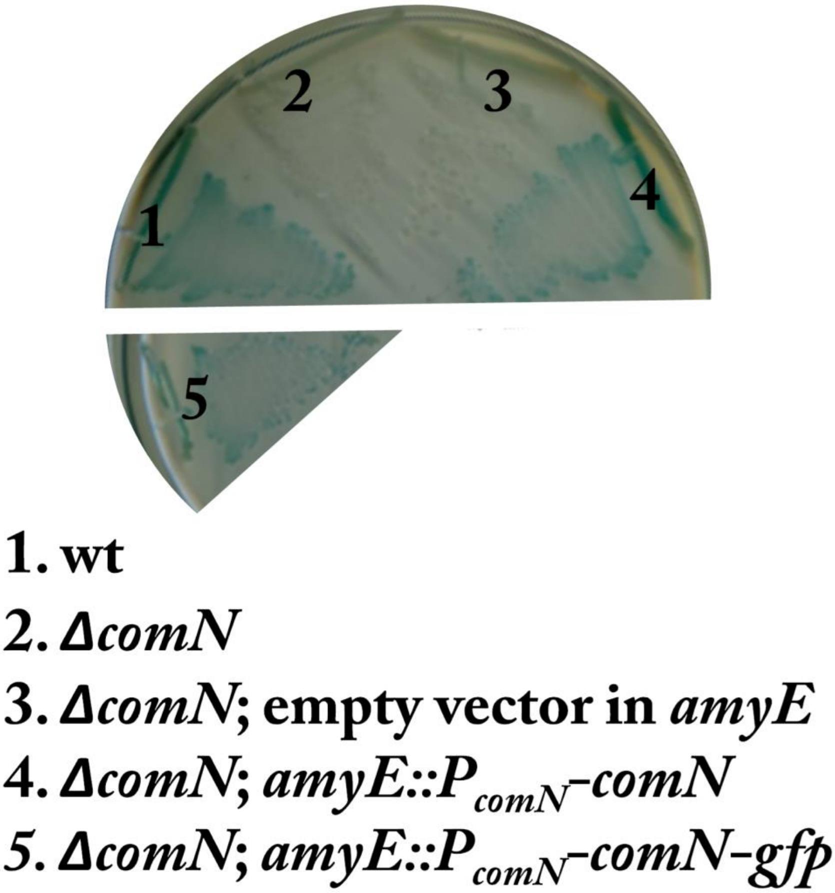

FIGURE S3

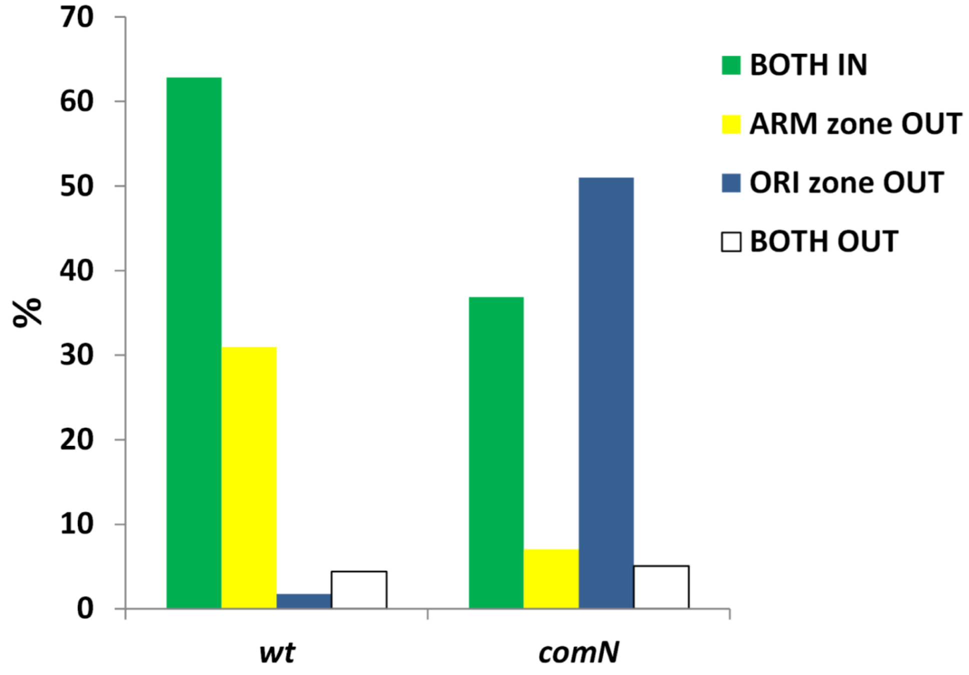
70
60
50
40
30
20
10
0
%
wt
comN
BOTH IN
ARM zone OUT
ORI zone OUT
BOTH OUT

FIGURE S4

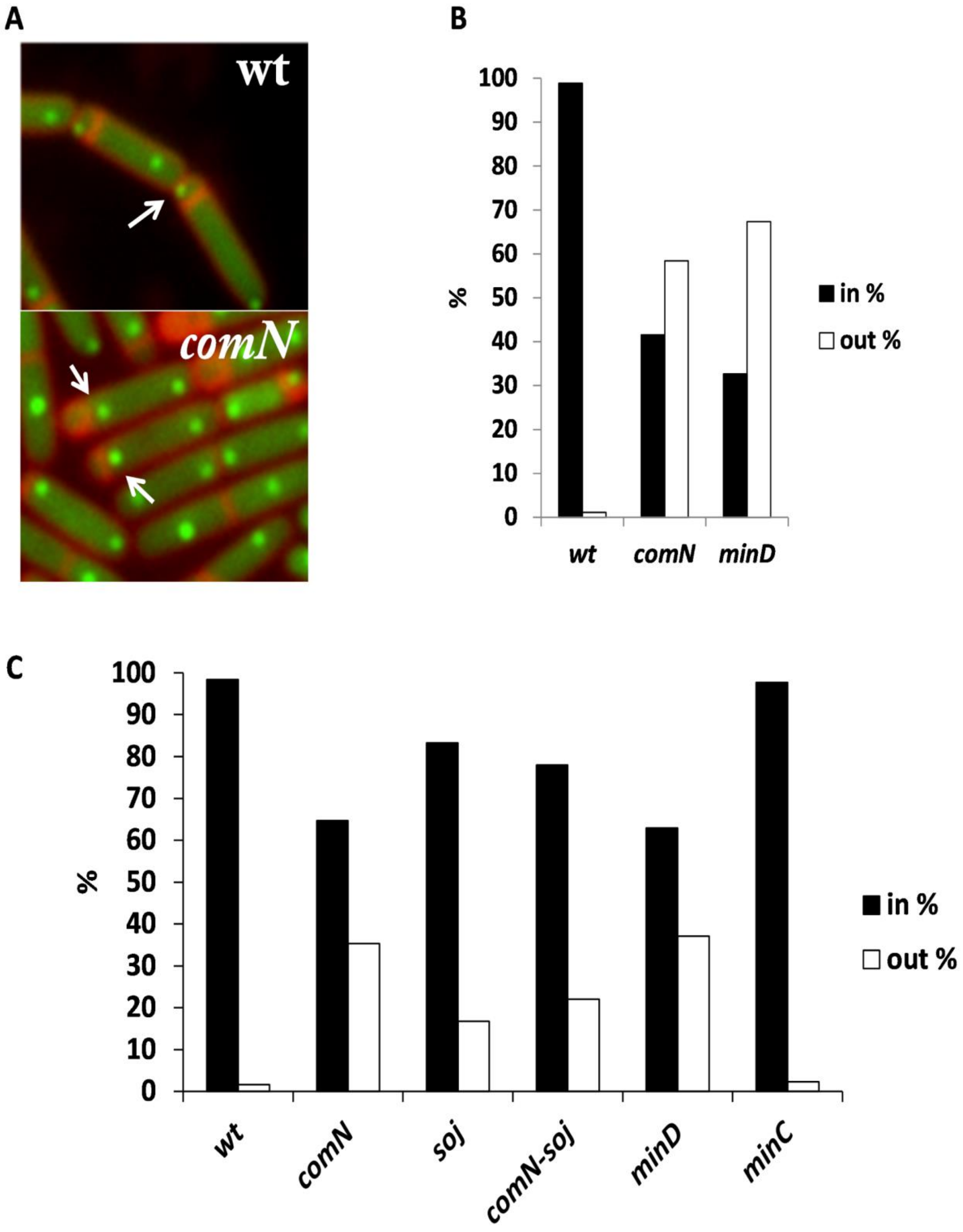
A
wt
comN
B
100
90
80
70
60
50
40
30
20
10
0
%
in %
out %
wt
comN
minD
C
100
90
80
70
60
50
40
30
20
10
0
%
in %
out %
wt
comN
soj
comN-soj
minD
minC

FIGURE S5

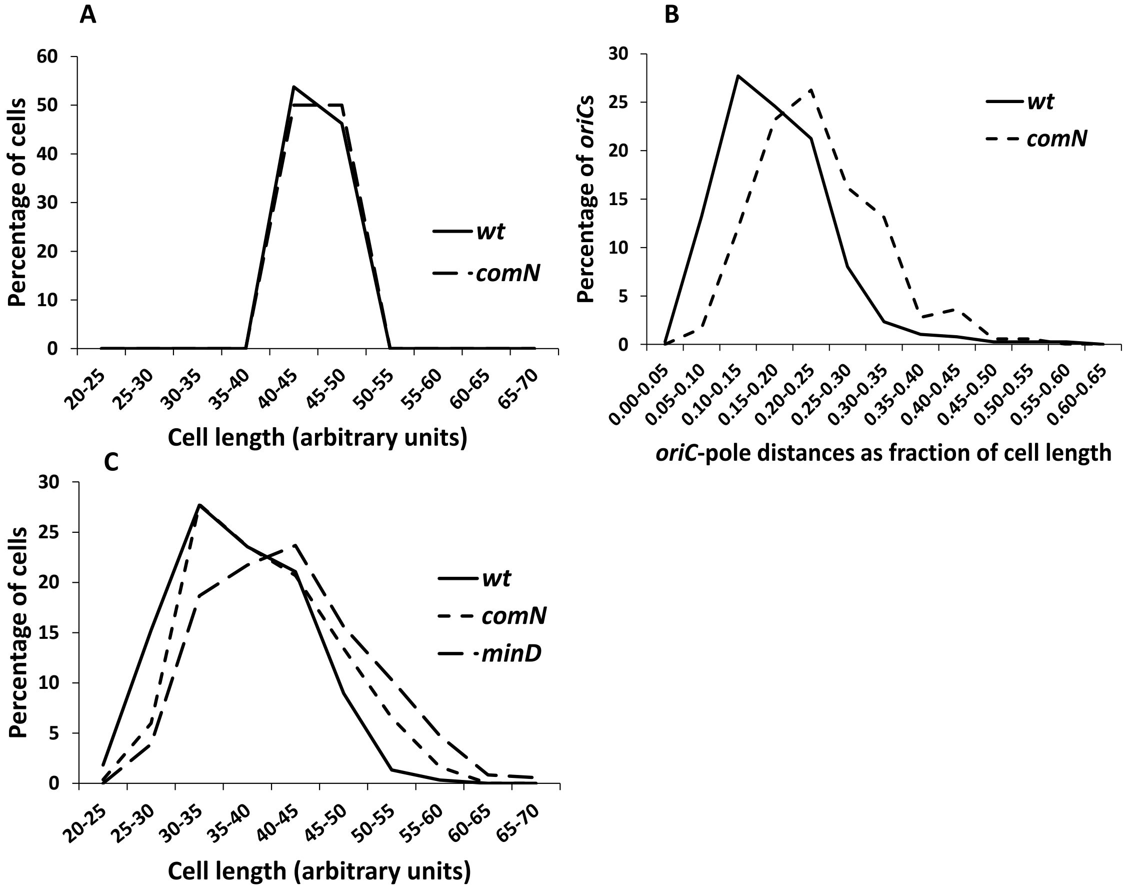

FIGURE S6

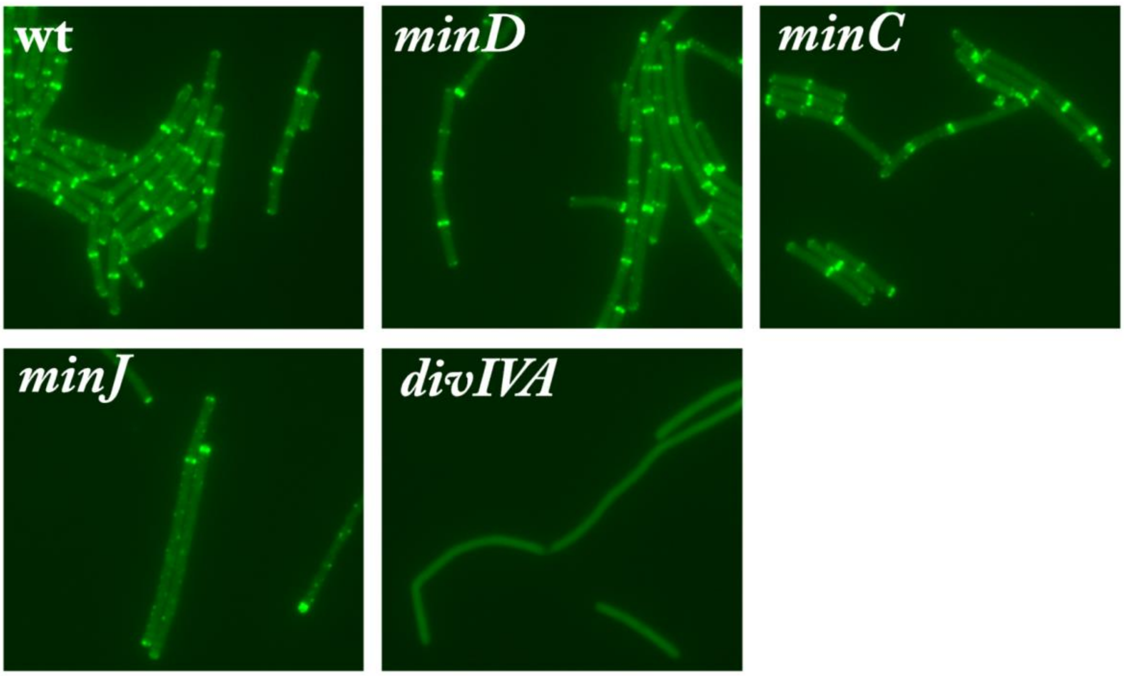

FIGURE S7

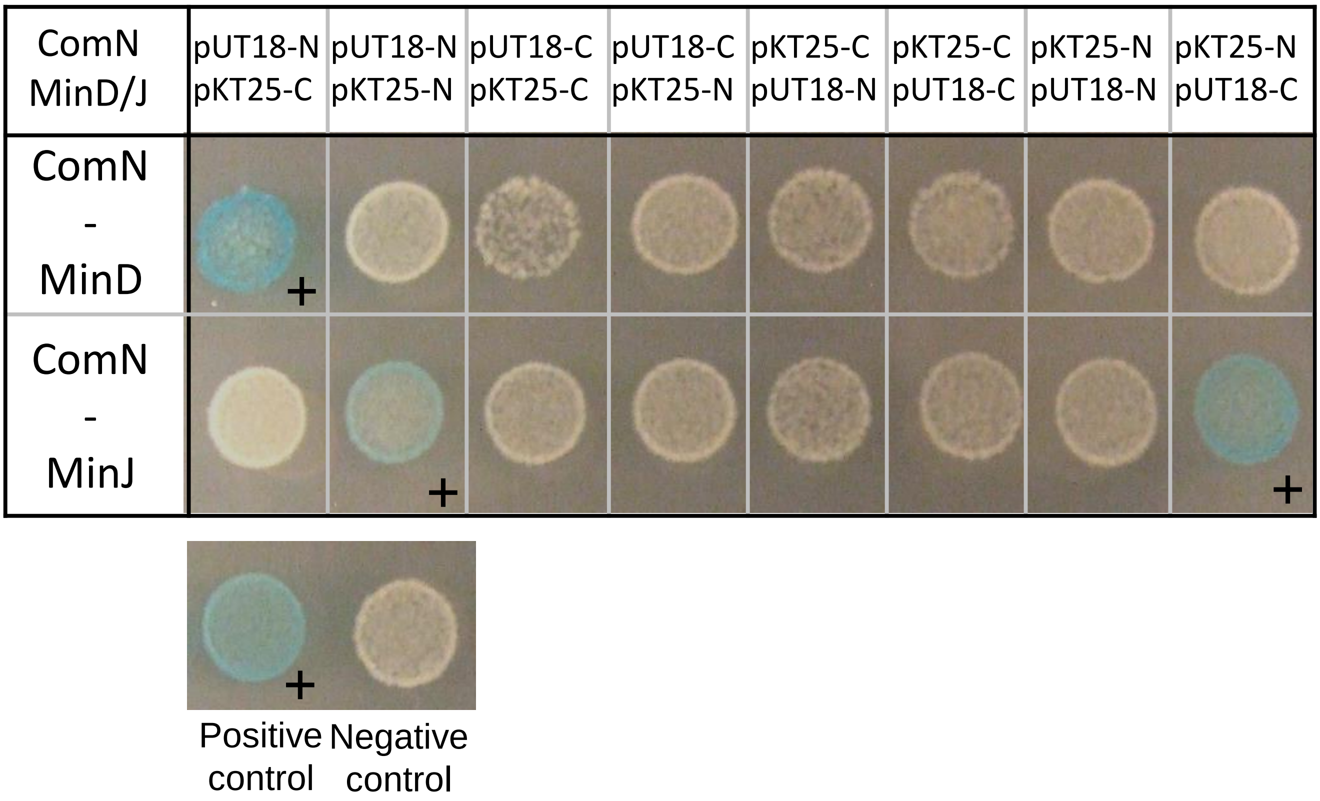
ComN MinD/J
pUT18-N pKT25-C
pUT18-N pKT25-N
pUT18-C pKT25-C
pUT18-C pKT25-N
pKT25-C pUT18-N
pKT25-C pUT18-C
pKT25-N pUT18-N
pKT25-N pUT18-C
ComN - MinD
ComN - MinJ
+
+
+
+
Positive control
Negative control
